# Comparative study of temperature effects on white sandstone and rockburst-like materials

**DOI:** 10.1371/journal.pone.0278782

**Published:** 2022-12-14

**Authors:** Zhengguo Zhu, Chaoyi Ma, Zhichun Fang, Weige Han, Renyuan Wang, Zhiming Han, Shaohu Chu, Yuanbo Gao

**Affiliations:** 1 State Key Laboratory of Mechanical Behavior and System Safety of Traffic Engineering Structures, Shijiazhuang Tiedao University, Shijiazhuang, Hebei, China; 2 Hebei Province Technical Innovation Center of Safe and Effective Mining of Metal Mines, Shijiazhuang, Hebei, China; 3 Key Laboratory of Roads and Railway Engineering Safety Control (Shijiazhuang Tiedao University), Ministry of Education, Shijiazhuang, Hebei, China; University of Vigo, SPAIN

## Abstract

Rockburst physical model test, as one of the important means to study deep tunnel engineering, reflects the main influencing factors of rockburst into the model test through similar theory, so as to reveal the formation mechanism, influencing factors and evolution law of different types of rockburst in deep tunnels. In order to study the mechanical properties of white sandstone in deeply buried tunnels at high ground temperatures, materials suitable for conducting rockburst physical and mechanical tests were developed on the basis of the Daqian Shi Ling tunnel project, and similar material ratios were preferentially selected on the basis of white sandstone. Judged by the rock burst propensity, similar materials with low strength and high brittle characteristics, can better simulate the characteristics of white sandstone, and all show a strong propensity to rock burst, is the ideal rock burst similar materials. Uniaxial compressive tests were conducted on similar materials and the original rock at different temperatures, and comparative analysis was performed. Through the study of stress, displacement and modulus of elasticity, it was concluded that the compressive strength of similar materials gradually increased with temperature in the range of 20–100°C, and the vertical displacement at peak strength decreased with increasing temperature. The damage forms of white sandstone and similar materials at different temperatures were comparatively analyzed, and it was obtained that the damage forms of white sandstone and similar materials were basically the same, with a few specimens showing tensile and shear damage, and most specimens showing the form of combined tensile and shear damage. The study of rock burst similar materials and the development of the failure characteristics of rock burst under the action of thermal coupling are of great significance to the mechanism of rock burst generation and prediction.

## Introduction

Deep hard rock tunnels are affected by high ground temperatures, which often induce high-intensity rock burst disasters during excavation. With the gradual expansion of the underground space, the temperature gradually increases, which can lead to changes in the internal results of the rock mass and affect its physical and mechanical behavior, thus posing a great threat to the safety of the underground space. However, it is relatively difficult to obtain the original rock to visualize the stress change and damage process of the rock mass. Rockburst physical model test, as one of the important means to study deep tunnel engineering, reflects the main influencing factors of rockburst into the model test through similar theory, so as to reveal the formation mechanism, influencing factors and evolution law of different types of rockburst in deep tunnels. Compared with indoor mechanical test, numerical simulation and field monitoring, the model test has the characteristics of easy operation, controllable main factors and rich and intuitive test results. For the physical model test, the nature of the model material characteristics have a great impact on the test results, the configuration of the material suitable for use in physical model tests is the basis for success. The key to this research is the formulation of materials suitable for rock blast physical and mechanical tests. The research method using similar materials is intuitive, simple, economical, and has a short test cycle; in addition, some of these parameters can be changed as needed, which is difficult to achieve under specific field conditions. Therefore, many scholars at home and abroad have studied materials similar to rockburst materials and the effect of high temperatures on rockbursts.

Xie et al. summarized the characteristics of the in-situ geological environment in the deep part of the Sichuan-Tibet Railway, revealed the engineering disturbance effect of deep surrounding rock disasters, and developed a surrounding rock quality classification method that can comprehensively reflect the influence of in-situ stress, ground temperature, and groundwater. Additionally, they introduced research ideas and prediction methods for the comprehensive prediction of rockburst in deep surrounding rocks [1]. Li et al. independently developed the physical test temperature field system and excavation device, and studied the characteristics of the rockburst subject to the effects of thermal–mechanical coupling on similar rockburst materials. They found that as the temperature increases, the greater the energy, the stronger the brittleness [2]. Hou et al. and others simulated similar materials (e.g., dolomite) in the deep mine, and used the orthogonal design principle to explore the model strength subject to different influencing factors. They summed up the empirical equation of similar material ratio based on a regression equation [3]. Zhou et al. and others formulated similar rockburst materials with low strength and high brittleness and studied the effects of different material contents on the brittleness and strength of the model [4]. With field tests, Yan et al. and others determined the damage form and spatial distribution of rockburst with high-geothermal temperature and high stress, and conducted rockburst prediction and analysis subject to different rockburst criteria [5–7]. Xu et al. prepared three similar rockburst materials [8]. Using dynamic impact tests and combined dynamic and static loading tests, they studied the influence of models with different characteristics on rockburst and found that dynamic and static combined loadings can considerably improve the compressive strength of the specimen. He et al. conducted an indoor rockburst simulation experiment on granite and studied the temperature field evolution of the unloading surface in the experiment [9]. The results showed that at the evolution stage of rock damage, the local temperature of the rock mass increased in conjunction with the development of cracks. Additionally, they measured the crack propagation process of the sample during the rockburst experiment by infrared thermal imaging technology. Wang et al. evaluated the degree of rockburst in the Dulongjiang Tunnel [10]. They selected quartz sand, cement, water, and glycerin as the raw materials of the tunnel rockburst model and analyzed the control parameters of the rockburst phenomenon and the disaster mechanism of the rockburst. On the basis of the analysis of the brittleness mechanism of inorganic materials, Dai et al. used the apparent elastic modulus to describe the brittleness characteristics of the deformation stage by adjusting the brittleness of similar materials [11]. They found that this parameter has the advantages of simple data processing and intuitive physical meaning. Driad-Lebeau et al. studied the deep coal seam subject to the influence of rockburst and conducted rockburst inversion through seismic conditions and in-situ stress data [12]. Their research revealed that the sandstone in the coal seam floor is exposed to high horizontal stress. Cardu et al. proposed a new formula for evaluating rockburst vibration attenuation and performed statistics and verification of vibration data for different rock types [13]. The formulation performs well and the derived formula is recommended when the measured data are available. Gomez-Rivas et al. studied the rock deformation process in earth materials, ranging from microscopic crystals to macroscopic tectonic plates, subject to the influence of temperature and explained how temperature controls the deformation mechanism and process of rocks [14]. Hans-Dieter Vosteen et al. studied rocks sampled at different crust depths, investigated the temperature dependence of thermal conductivity, heat capacity, and thermal diffusivity of different types of rocks, and laid a foundation for the study of the deformation properties of rocks at different temperatures [15]. Hirata et al. conducted tunnel stress analysis on the basis of the initial stress measurement of bedrock and adopted acoustic emission measurements in construction management to make the excavation safer [16]. Through comprehensive analysis of the obtained data, an effective method for safety control in the process of hard bedrock excavation was obtained. Keneti et al. proposed an index parameter to evaluate the severity of extreme brittle failure near complex underground caverns, thus providing guidance for minimizing strain shock hazards in geotechnical engineering design [17]. Rehbock-Sander Michael et al. used the software UDEC to dynamically analyze the rockburst process based on the field measured and calibrated rock parameters, and summarized the experience of rockburst prediction and mitigation [18]. Akdag et al. used acoustic emission and kinetic energy analysis to study the effects of thermal damage on the strain-type rockburst characteristics of brittle rock in true triaxial loading and unloading conditions [19]. They found that temperature caused microcracks, and with the increase of temperature, strain burst stress, initial rock ejection velocity, and kinetic energy gradually increased and exhibited stronger strain-burst behavior. Shcherbakov et al. obtained the time series of acoustic emission pulses during the generation of microcracks in granite specimens subjected to impact fracture at different temperatures [20]. They found that the damage accumulation kinetics and synergistic effects were quite different in the test temperature range of 20–600°C. Xu et al. prepared rockburst-similar materials composed of different proportions of aggregates, water-reducing agents, retarders, and other materials, and the most rock-burst-prone materials were selected from among them [21]. The geometric characteristics of the rock under different loading methods and opening positions were studied, and the occurrence mechanism of rockbursts was investigated. In order to explore the mechanism of strain-type rock burst, Li et al. used a similar model test method based on the elastic loading boundary to carry out an experimental study of strain-type rock burst induced by unloading of deep underground excavation [22]. The study shows that the proposed test method can better simulate the strain-type rock burst, which can provide test basis and data support for theoretical research and engineering practice of rock burst. Yang et al. conducted triaxial compression tests on saturated sandstone at different temperatures and different surrounding pressures [23]. The results show that freezing has a great influence on the mechanical properties of saturated sandstone, and based on five existing strength criterion theories, the Rocker strength criterion for saturated sandstone considering the temperature effect was proposed and verified to have good prediction effect based on the parameter calculation of test data. Zhang et al. proposed a method for assessing potential rockburst problems in underground engineering, taking into account the internal and external factors of rock mechanical properties, surrounding rock quality and ground stress, summarizing the geological conditions corresponding to rockbursts, and improving the test method of rockburst propensity index [24]. A new index of potential rock burst index is proposed, and the corresponding limit values are given, which can provide a simple and logical scientific assessment method for rock burst risk prediction at the engineering feasibility study stage. Based on a systematic summary and analysis of the advantages and shortcomings of domestic and foreign numerical methods for evaluating rockburst propensity, Qiu et al. proposed a new numerical index for evaluating rockburst propensity based on the basic theory of local energy release rate [25]. A comprehensive exposition of the construction of the index of the idea and the basic principle and its physical significance, to verify the new index of reasonableness and applicability. The study shows that the RERI index can indicate the risk area and location of high strain rock burst more accurately, and can relatively accurately indicate the extent of the excavation damage zone of deeply buried tunnels, providing new theoretical and analytical tools for the assessment and prevention of strain rock burst hazards and brittle damage in deeply buried tunnels. Liang et al. systematically summarized and classified long-term and short-term rockburst risk assessment methods, respectively, and analyzed the advantages and shortcomings of various existing assessment methods [26]. On this basis, the future development direction of long-term and short-term rockburst risk assessment is further proposed in order to improve the assessment of rockburst risk in hard rock.

The study of the mechanical properties and changes of rocks at different temperatures plays an important role in the development of engineering. Numerous scholars have studied the effects of temperature on rockburst of raw rock. Furthermore, some scholars controlled the temperature during the test. Most scholars heat the specimen in the drying box, but ignore the temperature environment in which the rock is broken. Therefore, this study sets the temperature in real time to study the effects of temperature on the tendency of the rockburst. Till date, the research on rockburst mechanisms is inadequate. In view of this, this study investigates the effects of different temperature conditions on rockburst-like materials and original rock and conducts real-time monitoring and measurements of temperature in the test for high accuracy results. The optimal ratio of materials similar to rockburst based on white sandstone was obtained experimentally, and the device used for testing was safe and simple. This provided a channel for studying rock compression tests at high temperatures and a more scientific and reasonable construction reference for similar working conditions. Overall, the findings of this study lay a foundation for studying the influence of deep underground temperature on rocks and for the prevention and control of rock bursts.

## Physical and mechanical properties of white sandstone

**Fig 1:**
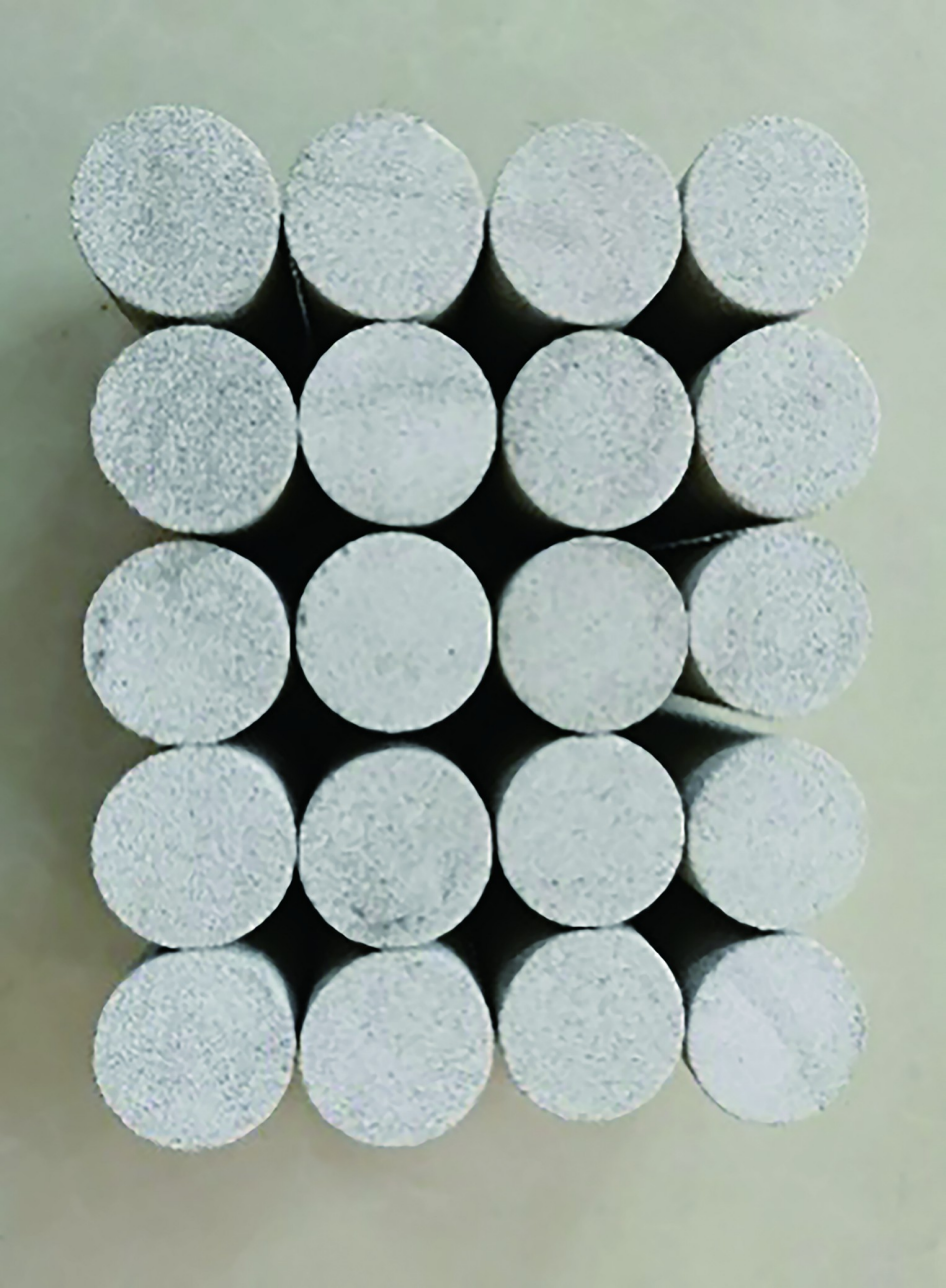
White sandstone specimens.

To obtain the physical and mechanical properties and proportions of materials similar to white sandstone, first, the physical and mechanical properties of the original white sandstone should be obtained. The white sandstone specimen is shown in Fig 1. Based on uniaxial compression and tensile tests, the physical and mechanical property parameters of white sandstone were obtained, as listed in Table 1.

**Table 1:** Physical and mechanical parameters of white sandstone.

Rock name	Compressive strength	Tensile strength	Elastic modulus
	*σ*_*c*_/MPa	*σ*_*t*_/MPa	*E*/GPa
**White sandstone**	29.76	6.14	7.41

The material tested comes from the Daqian Shiling Tunnel. The Daqian Shiling Tunnel is located 5km east of Jiangjiapuzi. The starting and ending mileage of the tunnel is DK69+225~DK71+685, and the total length of the tunnel is 2460m. According to geological survey and drilling, the internal distribution of the tunnel is mainly sandstone.

### Rockburst-like materials

To make the physical quantities of the white-sandstone-like material model achieve similar effects as the original white sandstone, an experiment was conducted based on the white sandstone according to the similarity theory. Thus, the optimal ratio of the white-sandstone-like material was obtained, and subsequently, the rockburst responses of the white-sandstone-like materials were studied.

#### Orthogonal design scheme of white-sandstone-like material model

This experiment was based on the orthogonal design scheme and four influencing factors were chosen, namely quartz sand (factor A), iron powder (factor B), gypsum cement ratio (factor C), and sand particle size (factor D). Four levels for each influencing factor were set; the levels of the four influencing factors are listed in Table 2. Thus, 16 experiments were conducted. The orthogonal design table used L16 (4^4^), as presented in Table 2.

**Table 2:** Design table of orthogonal test.

Group	A Quartz sand/%	B Iron power/%	C Plaster: cement	D Sand size/mm
**L1**	45	0.5	1:1	0.25–0.5
**L2**	45	1	1.5:1	0.5–1
**L3**	45	1.5	2:1	1–2
**L4**	45	2	3:1	2–4
**L5**	50	0.5	1.5:1	1–2
**L6**	50	1	1:1	2–4
**L7**	50	1.5	3:1	0.25–0.5
**L8**	50	2	2:1	0.5–1
**L9**	55	0.5	2:1	2–4
**L10**	55	1	3:1	1–2
**L11**	55	1.5	1:1	0.5–1
**L12**	55	2	1.5:1	0.25–0.5
**L13**	60	0.5	3:1	0.5–1
**L14**	60	1	2:1	0.25–0.5
**L15**	60	1.5	1.5:1	2–4
**L16**	60	2	1:1	1–2

Two types of cylindrical specimens with sizes of φ 50 mm × 100 mm were prepared using circular double-open plastic mold for compressive strength test, 16 groups of experiments are designed, and three specimens are prepared for each group of experiments, a total of 48 specimens. Cylindrical specimens with dimensions of 50 mm × 50 mm were prepared for the Brazilian splitting test,16 sets of tests are designed, and each set of tests prepares three test pieces, a total of 48 test pieces.

**Fig 2:**
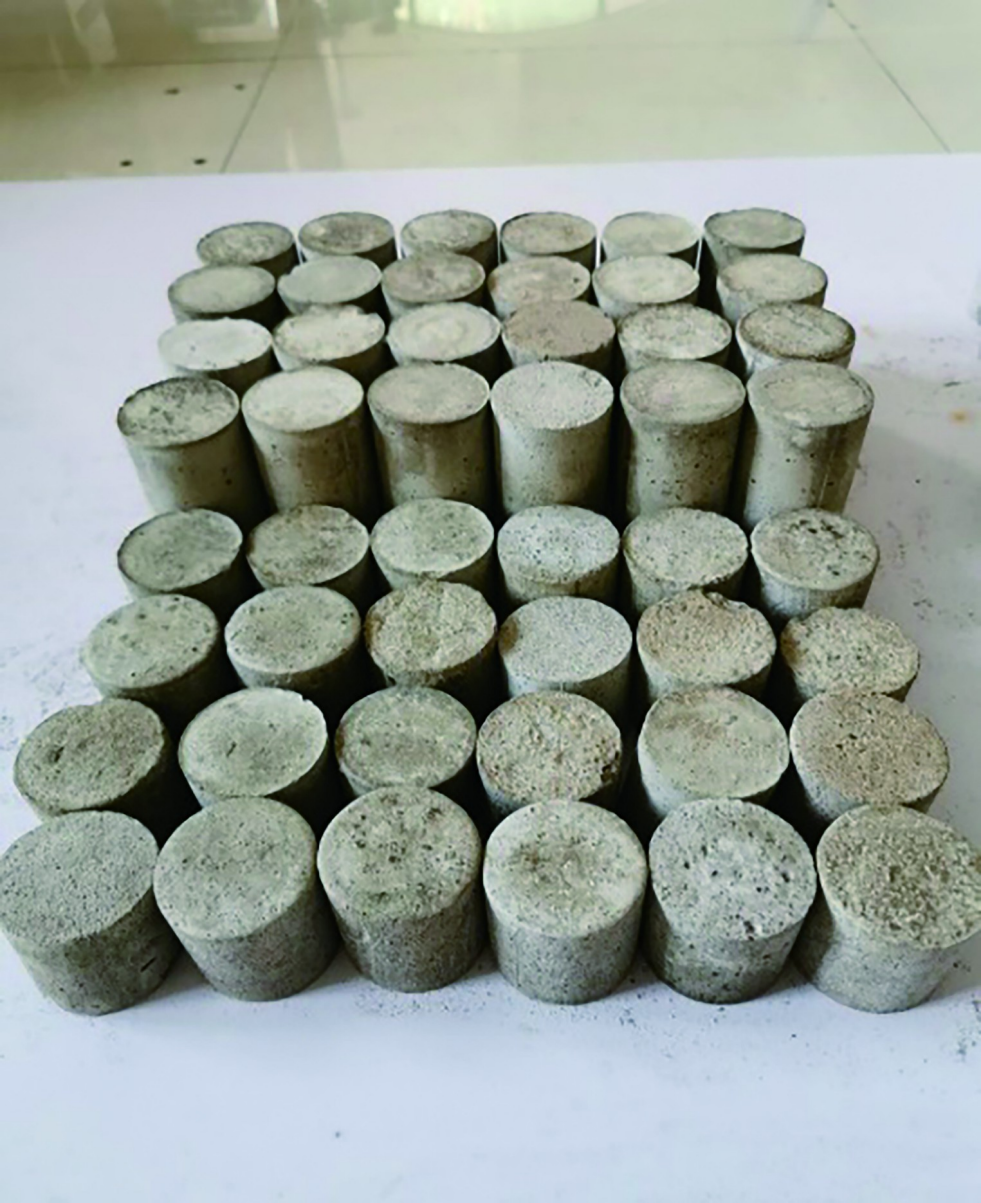
Standard specimens.

**Fig 3:**
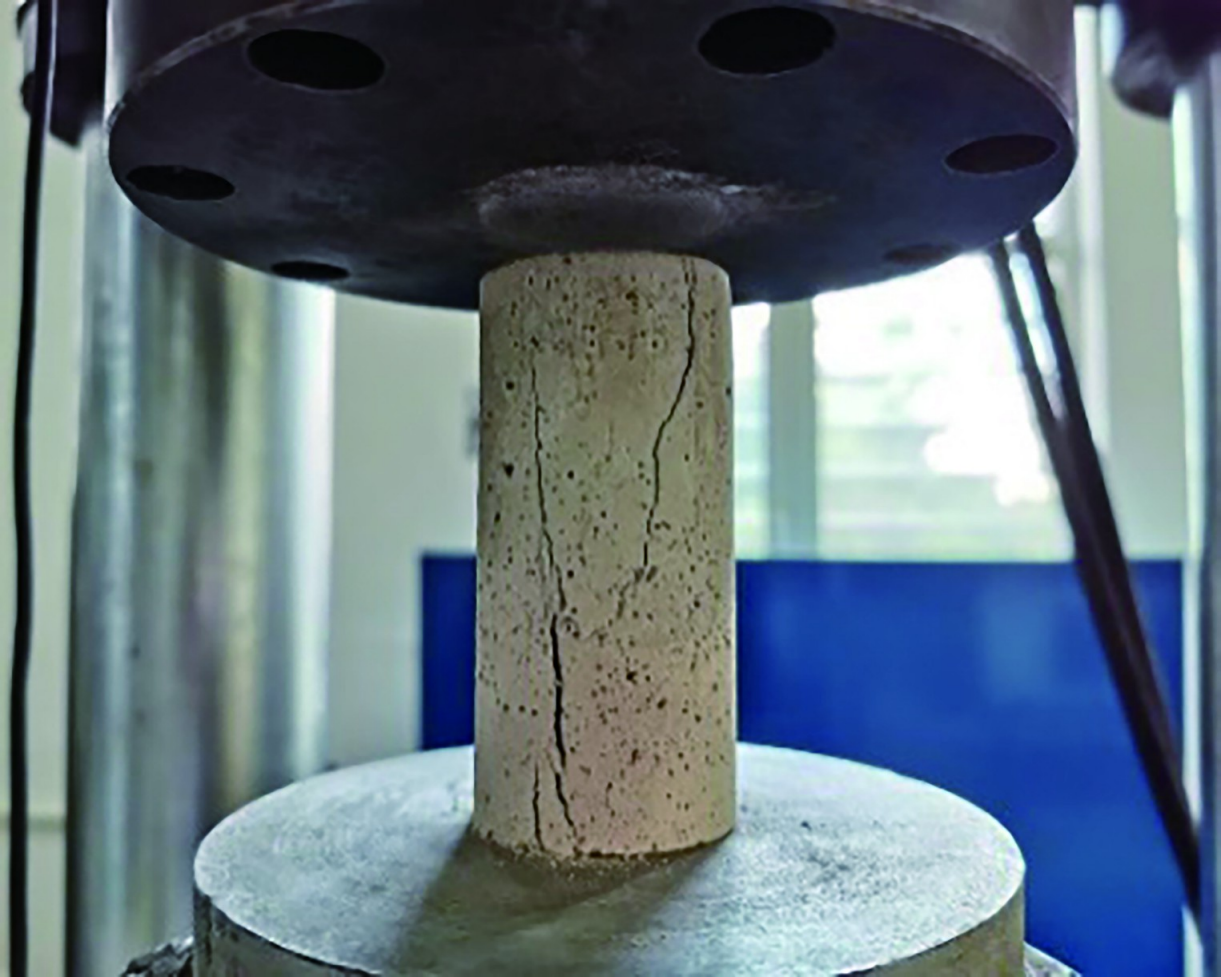
Compressive strength test for rockburst-like materials.

**Fig 4:**
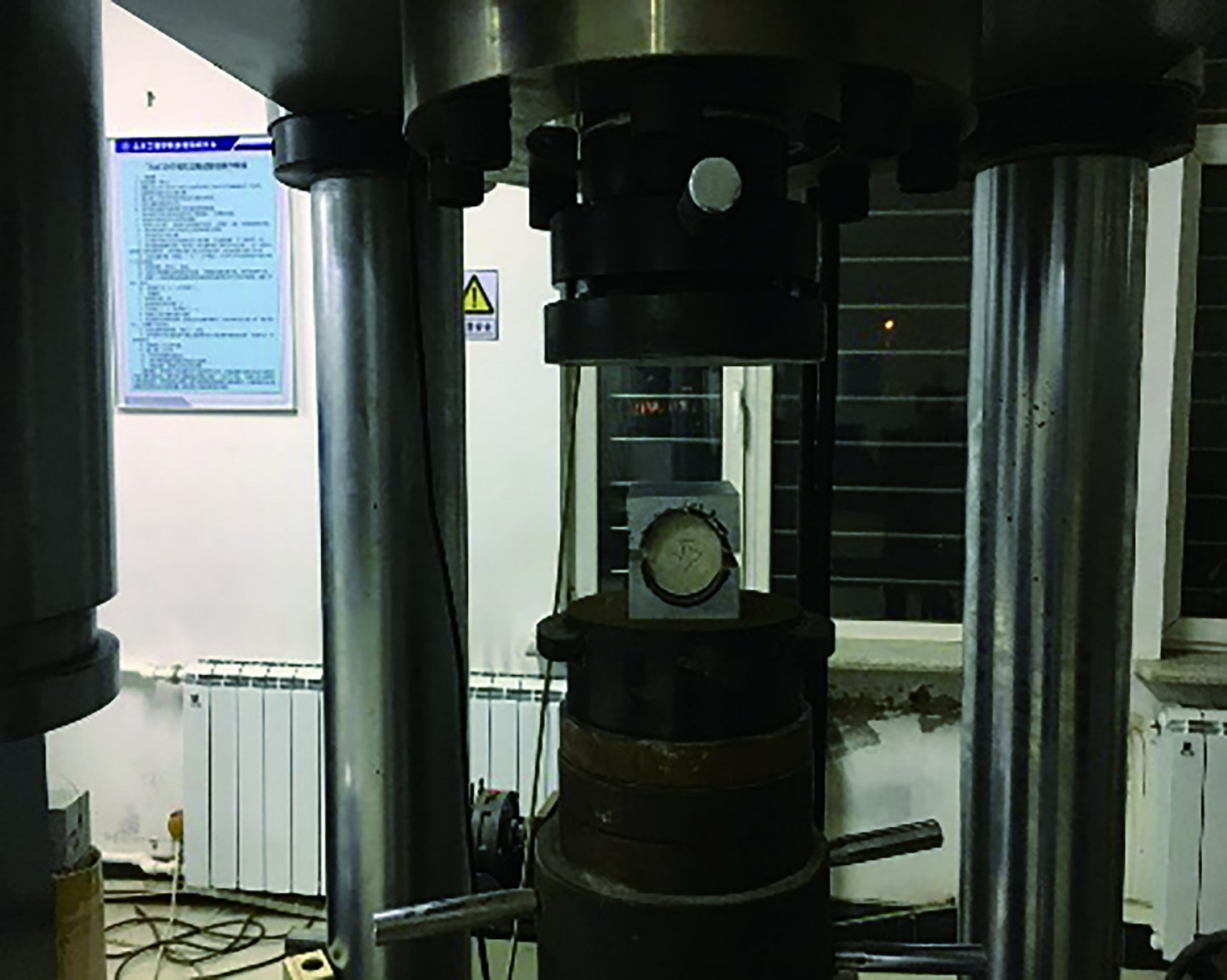
Tensile strength test for rockburst-like materials.

According to the production rules, the allowable range of deviation between two end faces was ±0.05 mm, and the allowable range of vertical deviation was 0.25°. The specimen obtained is shown in Fig 2. Considering the low strength and high brittleness specimen characteristics, the loading speed was set to 0.1 MPa/s until the specimen failed. The test process is shown in Figs 3 and 4, respectively.

According to the uniaxial compressive strength and tensile tests, the compressive strength, tensile strength, elastic modulus and compressive-tensile ratio of the specimens were obtained, and the orthogonal ratio test results are listed in Table 3.

**Table 3:** Test results of orthogonal ratio.

Group	Compressive strength *σ*_*c*_ /MPa	Tensile strength *σ*_*t*_ /MPa	Elastic modulus *E*/GPa	Ratio of compressive to tensile strengths *σ*_*c*_*/σ*_*t*_
**L1**	14.14	2.73	2.03	5.18
**L2**	13.00	2.76	1.81	4.71
**L3**	13.50	2.47	1.57	5.46
**L4**	9.94	2.09	2.86	4.76
**L5**	13.59	2.85	2.16	4.77
**L6**	8.83	2.33	2.23	3.79
**L7**	12.15	2.44	1.65	4.98
**L8**	11.50	2.37	1.74	4.85
**L9**	12.47	2.23	2.07	5.59
**L10**	12.88	2.48	1.72	5.19
**L11**	10.93	2.62	1.96	4.17
**L12**	9.30	2.12	1.53	4.39
**L13**	13.29	2.24	1.82	5.93
**L14**	9.94	2.82	1.84	3.52
**L15**	9.79	2.16	1.76	4.53
**L16**	8.96	2.01	1.96	4.46

Range analysis is a commonly used method in orthogonal experimental design. In this study, sensitivity analysis was conducted for each influencing factor based on the concept of range. Range analysis can reflect the degree of dispersion between data simply and directly. The extreme difference indicates that the influence factor has an obvious influence on the research index, and vice versa is not obvious.

The calculation method of the range analysis method is shown in Eq (1).

$$R=I_{max}-I_{min},$$ (1)

where *R* is the range; and *I*_*max*_ and *I*_*min*_ are the maximum and minimum values of the average factor, respectively.

Combining Tables 2 and 3 and considering the influences of factor C at level 2 as an example, the compressive strength values of tests 2, 5, 12, and 15 are involved, and the average value is (4.71 + 4.77 + 4.39 + 4.53) / 4 = 4.60.

**Fig 5:**
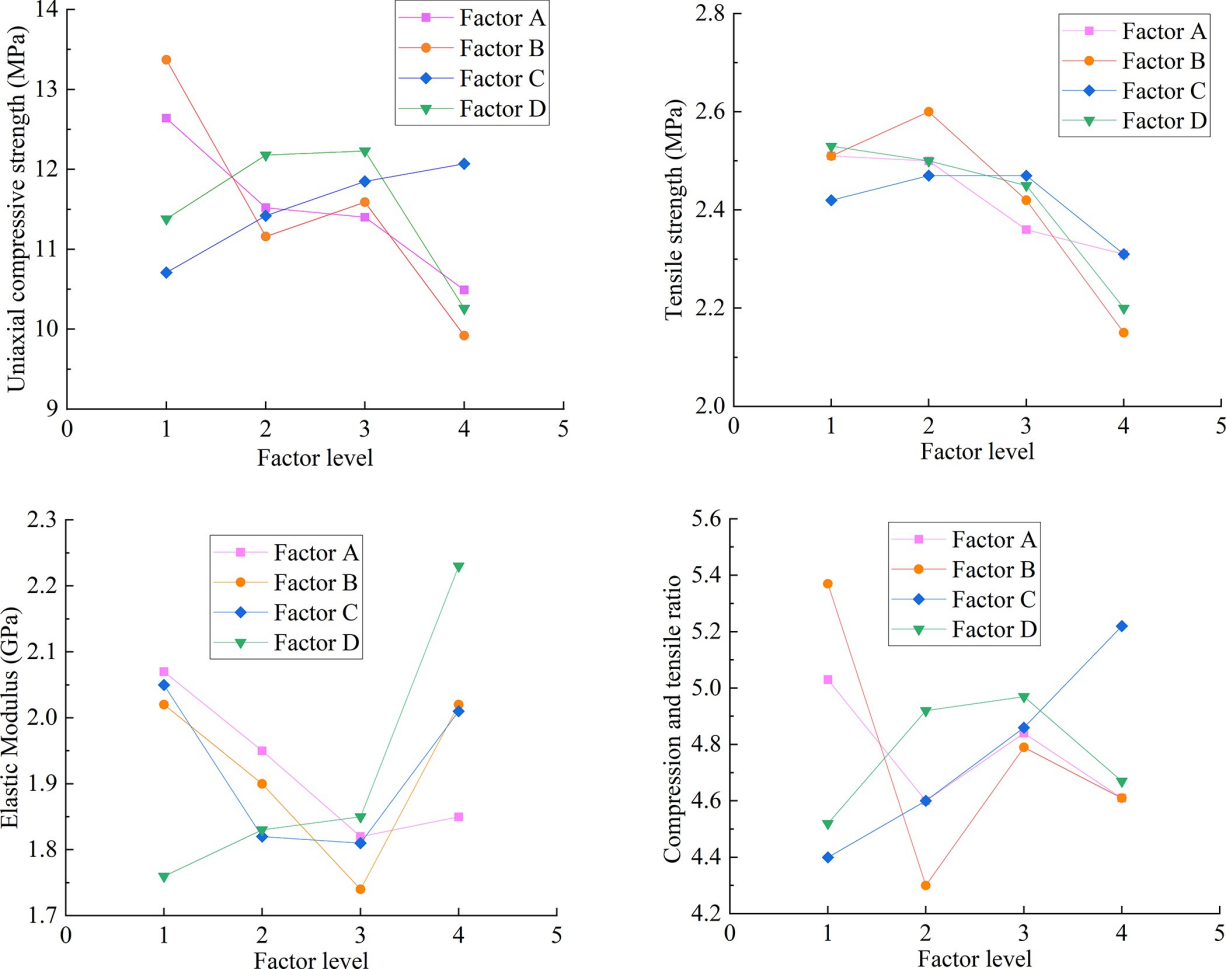
Main effects of range analysis. (a) Influences of various factors on compressive strengths of rockburst-like materials. (b) Influences of various factors on tensile strengths of rockburst-like materials. (c) Influences of various factors on the elastic moduli of rockburst-like materials. (d) Influences of various factors on the compressive tensile ratios of rockburst-like materials.

The mean and range of the influence of each influencing factor on the compressive and tensile ratios at different levels are listed in Table 4 and shown in Fig 5(D).

**Table 4:** Range analysis of *σ*_*c*_*/σ*_*t*_.

Factor level	A Quartz sand/%	B Iron power/%	C Plaster: cement	D Sand size/mm
**I**_**1**_	5.03	5.37	4.40	4.52
**I**_**2**_	4.60	4.30	4.60	4.92
**I**_**3**_	4.84	4.79	4.86	4.97
**I**_**4**_	4.61	4.61	5.22	4.67
**Range**	0.43	1.07	0.72	0.45

Table 4 shows that factor B yields the largest range of compressive and tensile ratios, reaching a value of 1.07. Accordingly, these outcomes indicate that factor B has the most obvious influence on σ_c_/σ_t_ and plays a leading role. Based on range ranking, the degree of influence of each factor on the compressive and tensile ratio is ranked in the following order: B > C > D > A.

Fig 5 lists the main effects of range analysis of the influence of various factors on uniaxial compressive strength, tensile strength, elastic modulus, and compressive–tensile ratio.

Fig 5(A) shows that the influence of each factor on the uniaxial compressive strength of similar materials is in the order of A > D > B > C. With increase in quartz sand (factor A), the uniaxial compressive strength exhibited a decreasing trend. With increase in gypsum–cement ratio (factor C), the uniaxial compressive strength exhibited a gradually increasing trend. This shows that the content of quartz sand (factor A) plays a leading role in controlling the uniaxial compressive strength σ_c_ of similar materials.

Fig 5(B) shows that the influence of each factor on the tensile strengths of similar materials is in the order of B > D > A > C. With increase in quartz sand (factor A) and sand particle size (factor D), the tensile strength exhibited a decreasing trend. With increase in iron powder content (factor B), the tensile strength first increases and then decreases abruptly. There is no specific rule to follow for the effect of the gypsum–cement ratio (factor C) on tensile strength. This shows that the content of iron powder (factor B) plays a major role in controlling the tensile strengths σ_t_ of similar materials of white sandstone.

Fig 5(C) shows that the influence of each factor on the elastic modulus of similar materials is in the order of D > B > A > C. With increase in sand particle size (factor D), the elastic modulus exhibited a gradually increasing trend. This shows that the sand particle size (factor D) plays a dominant role on the elastic modulus E for cases wherein similar materials are considered.

Fig 5(D) shows that the influence of each factor on the compressive and tensile ratios of similar materials is in the order of B > C > D > A. With increase in the gypsum cement ratio (factor C), the compressive and tensile ratios of similar materials tend to increase. No specific rule exists that needs to be followed for the effect of quartz sand content (factor A) on the compressive and tensile ratios of similar materials. This shows that the main factor controlling the compressive tensile ratio of similar materials is the iron powder content (factor B).

According to the test results, various mechanical parameters (uniaxial compressive strength, tensile strength, elastic modulus, compressive–tensile ratio), and influencing factors (quartz sand content, iron powder content, gypsum–cement ratio, sand particle size) were fitted with Origin 2021b, and a multiple linear regression equation was established using Eqs (2)–(5).

$$\sigma_{c}=20.47191‐13.15295x_{1}‐198.1285x_{2}+0.64007x_{3}‐0.41513x_{4},$$ (2)

$$\sigma_{t}=3.81755‐1.5x_{1}‐25.4x_{2}‐0.06229x_{3}‐0.09374x_{4}$$ (3)

$$E=2.52789‐1.585x_{1}‐3.15x_{2}+0.009x_{3}+0.13126x_{4},\;\mathrm{and}$$ (4)

$$\sigma_{c}/\sigma_{t}=5.495‐2.0222x_{1}‐35.0552x_{2}+0.41225x_{3}+0.00348x_{4,}$$ (5)

where *σ*_*c*_ and *σ*_*t*_ are the compressive and tensile strengths (in MPa), respectively; *E* is the elastic modulus (in GPa); *σ*_*c*_/*σ*_*t*_ is the tensile and compressive ratio; and *x*_1_, *x*_2_, *x*_3_, and *x*_4_ are the contents of quartz sand, iron powder, gypsum–cement ratio, and sand particle size, respectively.

According to the physical and mechanical parameters of the original rock presented in Table 1 combined with the standard formula selected for similar materials [27–29], the mechanical parameters of the similar material specimens are summarized in Table 5.

**Table 5:** Similar mechanical parameters of materials.

Rock name	Compressive strength *σ*_*c*_/MPa	Tensile strength *σ*_*t*_/MPa	Elastic modulus *E*/GPa
**Rockburst-like material**	11.50	2.37	2.86

The optimal ratio of similar materials calculated by Eqs (2)–(5) is listed in Table 6. Based on the physical and mechanical parameters of the original rock in Table 1, combined with the criteria for the selection of similar materials in Eq (6), brought into the equation and calculated the Table 6.

**Table 6:** Multiple linear regression was used to determine the ratio of similar materials.

Similar material	A Quartz sand/%	B Iron powder/%	C Plaster: cement	D Sand size/mm
**Ratio**	36	1.9	1.8:1	2~4

The main criteria for selecting similar materials are as follows:

$$\alpha =\frac{l_{m}}{l_{P}},\;\beta =\frac{\gamma_{M}}{\gamma_{P}},\;\sigma_{c}^{M}=\sigma_{c}\alpha \beta ,$$

$$E^{M}=E\alpha \beta ,\;\sigma_{t}^{M}=\sigma_{t}\alpha \beta ,\;\mu^{M}=\mu ,$$ (6)

where l_p_ is the original rock size (mm), l_m_ is the size of the similar model specimens (mm), α is the geometric similarity coefficient, β is the gravity similarity coefficient, γ_P_ is the natural gravity of white sandstone (KN/m^3^), γ_M_ is the dry weight of the similar material model (KN/m^3^), σ_c_ is the uniaxial compressive strength of the original rock (MPa), $\sigma_{c}^{M}$ is the uniaxial compressive strength of the similar material model (MPa), *E* is the elastic modulus of the original rock (GPa), *E*^M^ is the elastic modulus of the similar material model (GPa), μ is Poisson’s ratio of the original rock, and μ^M^ is Poisson’s ratio of the similar material model.

The results show that the physical and mechanical properties of similar materials conform to the physical and mechanical properties of white sandstone when the optimal ratio of similar materials yields the following characteristics: quartz sand content 36%, iron powder content 1.9%, gypsum cement ratio 1.8:1, and sand particle size in the range of 2–4 mm.

#### Evaluation of rockburst tendency

**Fig 6:**
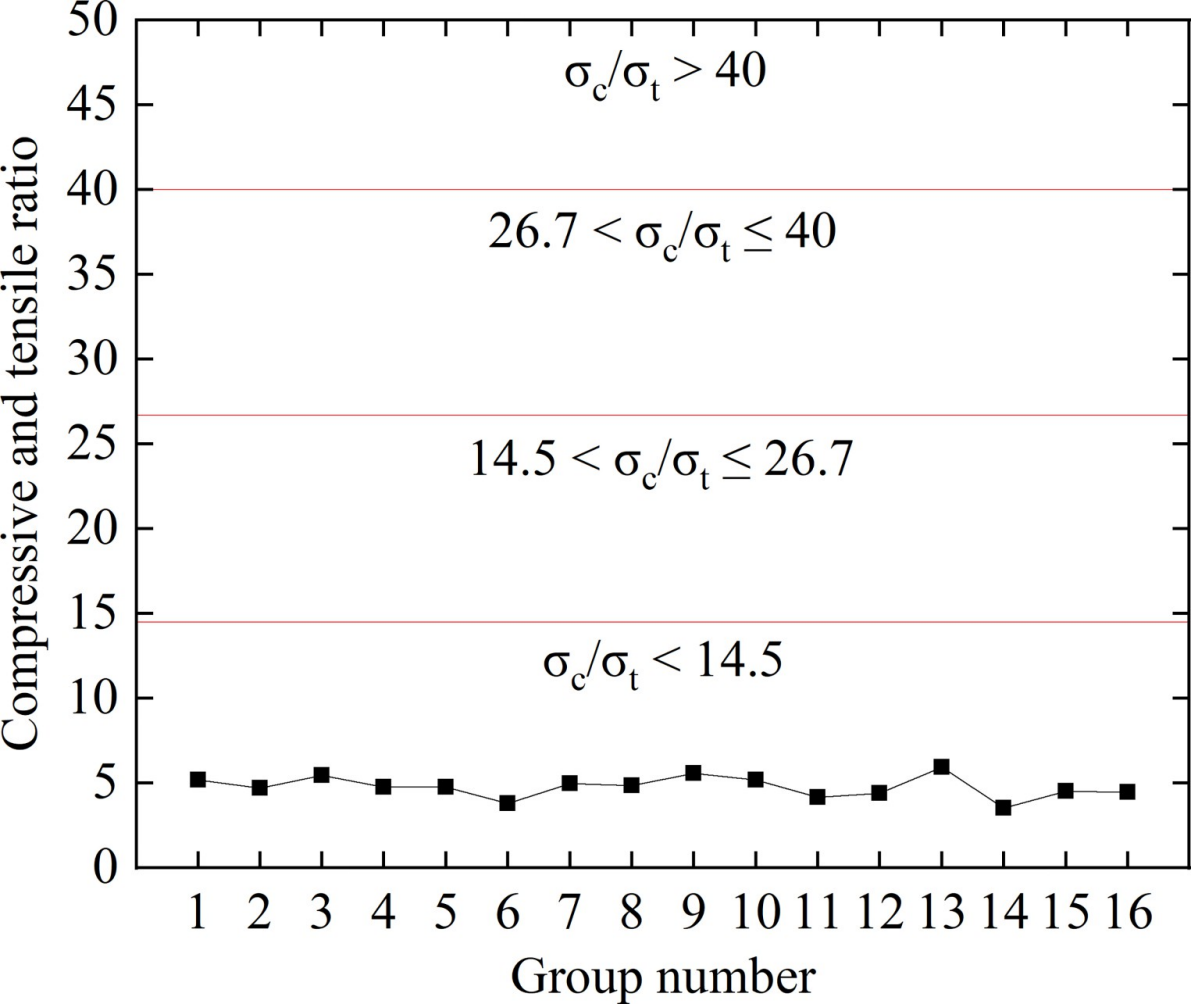
Evaluations of rockburst tendencies based on compression and tensile ratio.

**Fig 7:**
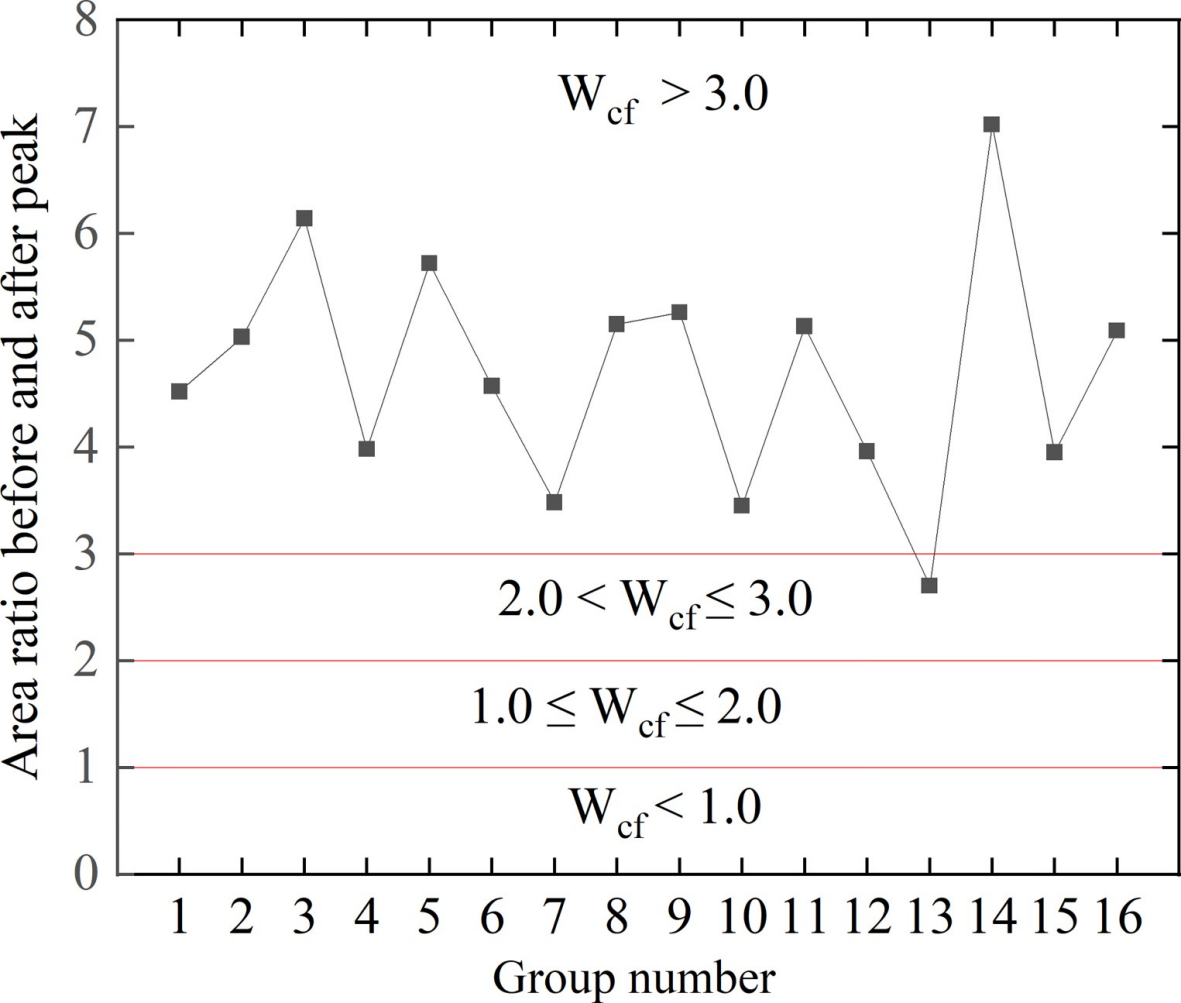
Determination of rockburst tendencies based on the area ratio before and after peak.

The Figs 6 and 7 show the discrimination results of rockburst propensity of 16 groups of similar white sandstone materials.

(1) The compressive and tensile ratio represents the brittleness of the rock and can be used to measure the rockburst tendency of the material. Evaluation of the rockburst tendency according to the brittleness characteristics of the rock is a commonly used method. The general concept based on which the evaluation outcome was formulated is described as follows.

When σ_c_/σ_t_ < 14.5, the material has strong rockburst tendency; when 14.5 < σ_c_/σ_t_ ≤ 26.7, it has moderate rockburst tendency; when 26.7 < σ_c_/σ_t_ ≤ 40, it has weak rockburst tendency, and when σ_c_/σ_t_ > 40, there is no rockburst tendency.

The compressive tensile ratio and rockburst tendency assessment diagram of the 16 groups of similar materials evaluated herein in the orthogonal test are shown in Fig 6.

(2) Assessing the rockburst tendency of similar materials based on the area ratio of the stress–strain curve before and after the peak value is expressed by formula (7) [30–32],

$$W_{cf}=F_{1}/F_{2},$$ (7)

where *F*_1_ and *F*_2_ are the areas before and after the peak, respectively. Note that W_cf_ > 3.0 indicates a strong rockburst tendency; 2.0 < W_cf_ ≤ 3.0 indicates a moderate rockburst tendency; 1.0 ≤ W_cf_ ≤ 2.0 indicates a weak rockburst tendency, and W_cf_ < 1.0 indicates no rockburst tendency.

The assessment of the rockburst tendency by the area ratio before and after the peak is shown in Fig 7.

The rockburst tendency of the 16 groups of white sandstone-like materials was evaluated using the discriminating rockburst tendency method according to the index verification. The evaluation revealed that similar materials can emulate the characteristics of white sandstone more effectively. All of these materials exhibited strong rockburst tendencies and are ideal rockburst-like materials.

### Uniaxial compression test of materials similar to rockburst at different temperatures

#### Specimen preparation and maintenance

The optimal ratio in this experiment was selected and six groups of specimens were constructed. Uniaxial compression tests were conducted on rockburst-like materials at different temperature conditions. The specific preparation process is as follows.

**Fig 8:**
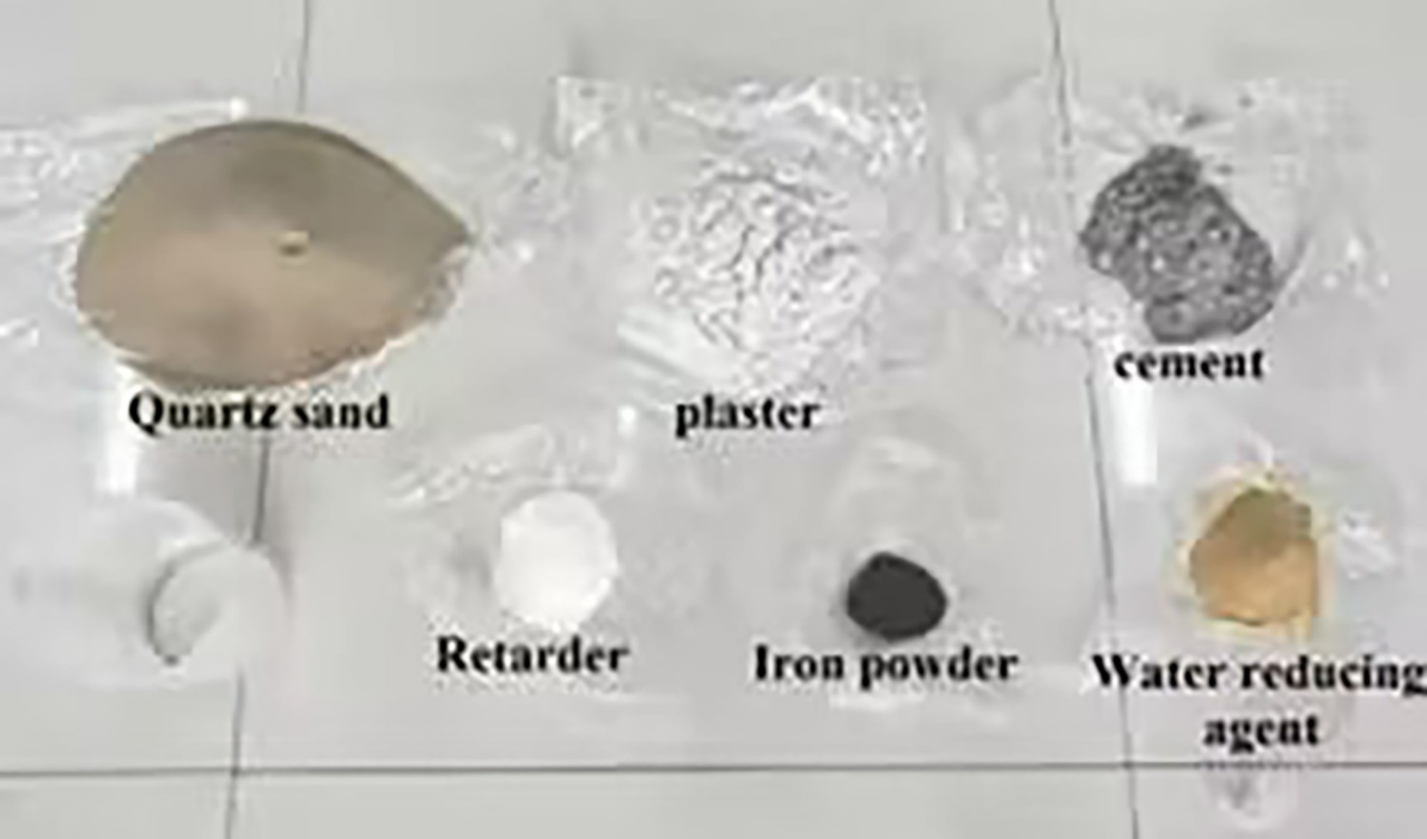
Similar material model of the test material covered by the study.

**Fig 9:**
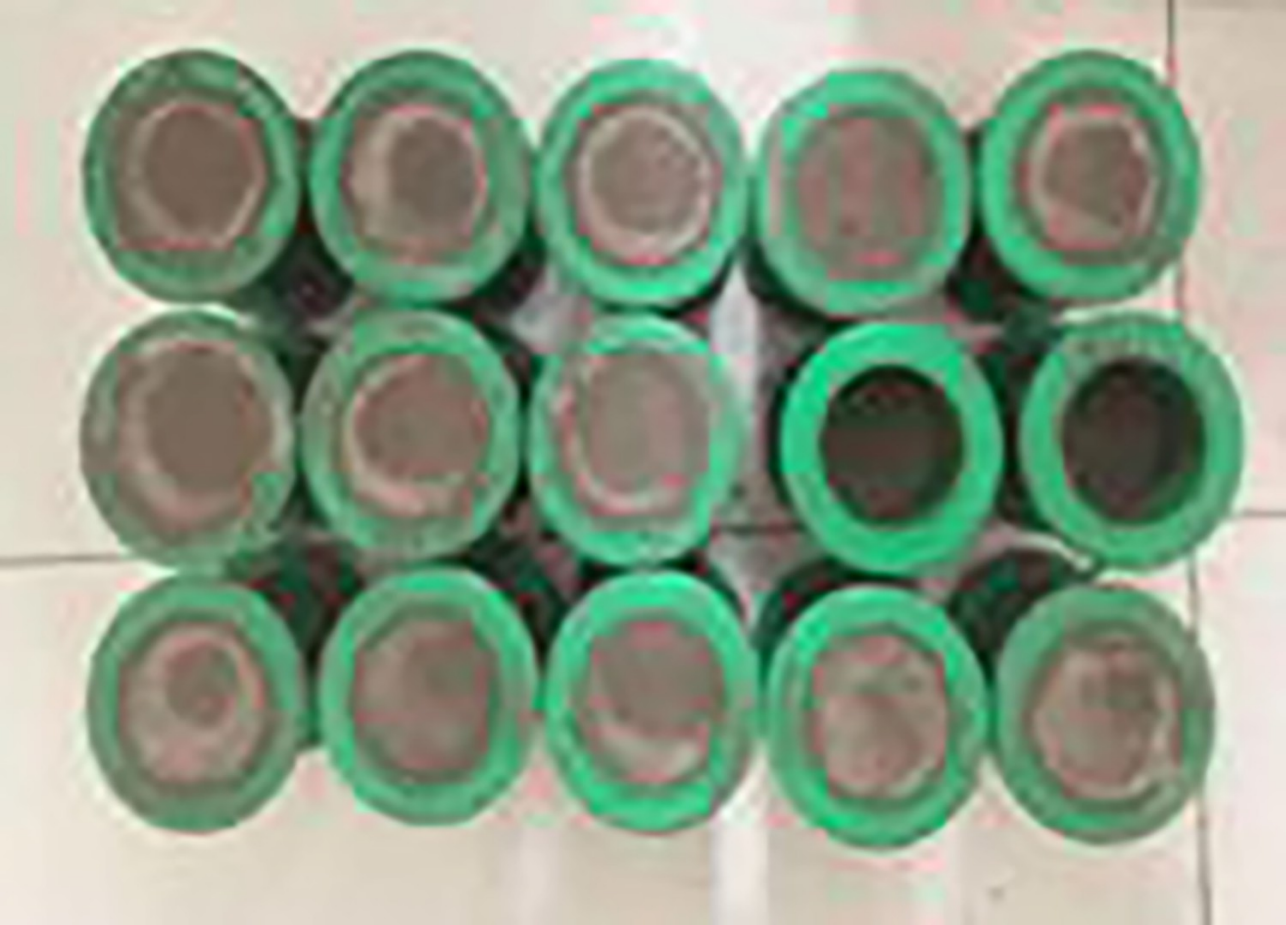
Sample preparation of similar materials covered by the study.

**Fig 10:**
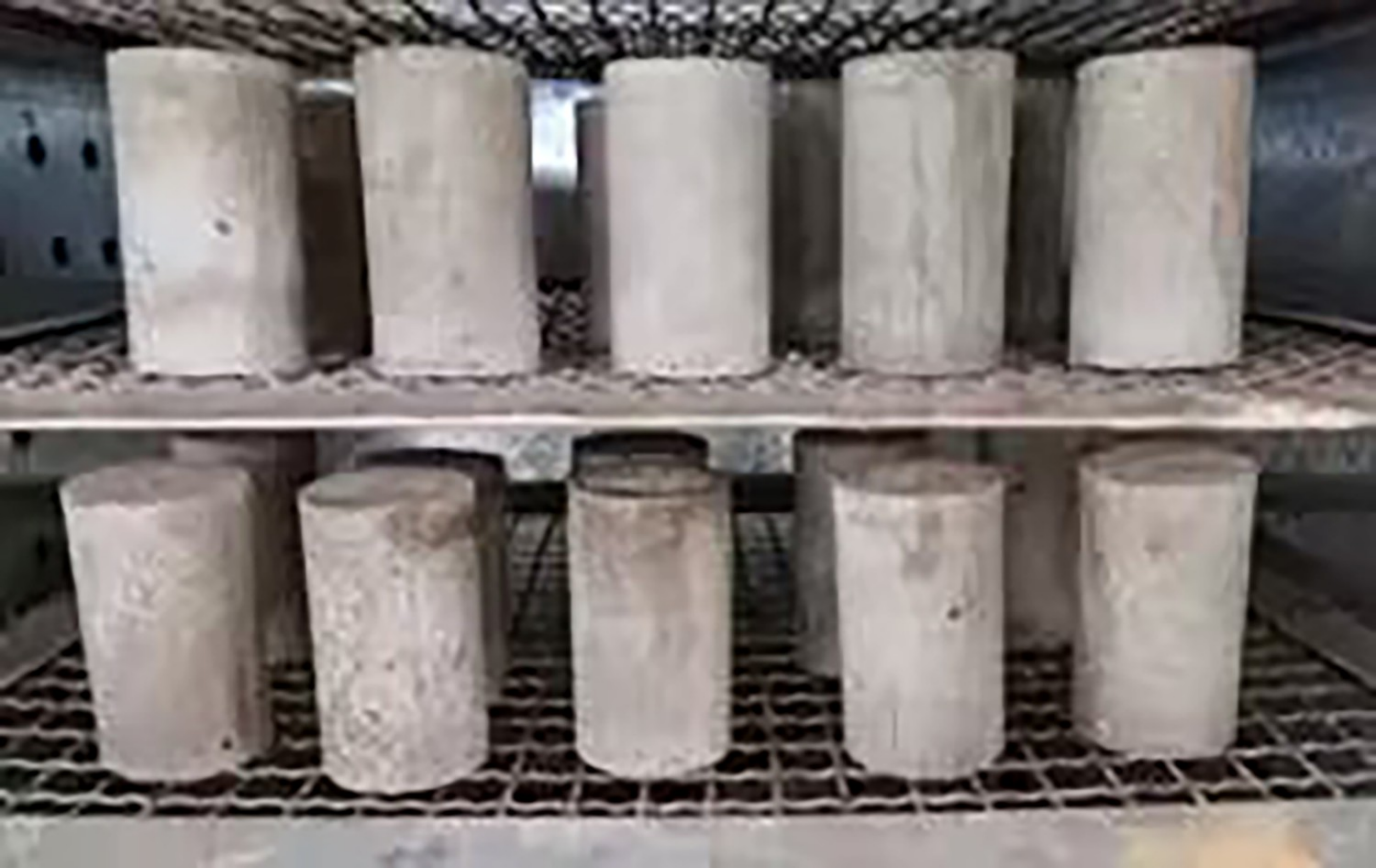
Maintenance of the specimens covered by the study.

**Fig 11:**
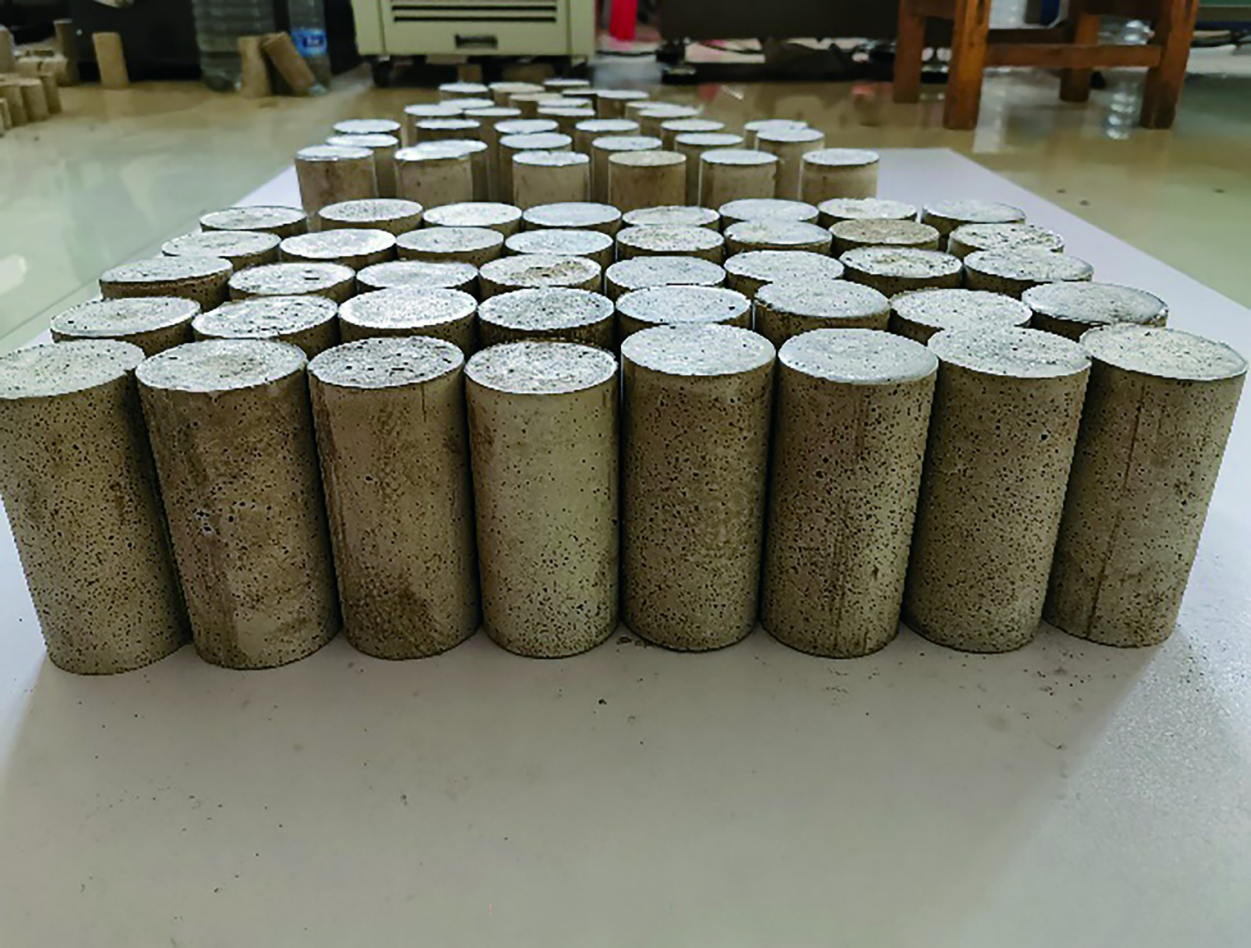
Rockburst-like materials specimens.

1. The raw materials were prepared; these primarily include quartz sand, iron powder, gypsum, cement, retarder, and water reducer.
2. The quartz sand was screened and 2–4 mm quartz sand was selected for testing.
3. According to the inferences of the orthogonal test, quartz sand content of 36%, iron powder content of 1.9%, and gypsum–cement at a ratio of 1.8:1 were prepared. Subsequently, retarder (1%) and water-reducing agent (1%) were added, and water mixing fully take reserve, as in Fig 8.
4. The weighed and measured materials were mixed evenly, poured into an oil-coated mold, and struck in layers with a rubber hammer to ensure that the test piece is dense. The sample preparation of similar materials involved in the study is shown in Fig 9.
5. The poured model was maintained under natural conditions, and the mold was removed after 24 h. The similar material test blocks were placed into the steam curing box for 28 d and subsequently, the mechanical test was conducted. The specimens covered by the maintenance study are shown in Fig 10. The sample of similar materials is shown in Fig 11.

#### Test method

After the rockburst-like material specimens were prepared, the test was conducted after the 28 d curing period expired. In this test, the model was subjected to uniaxial compression tests at different temperatures [33]. The specific test steps are as follows.

**Fig 12:**
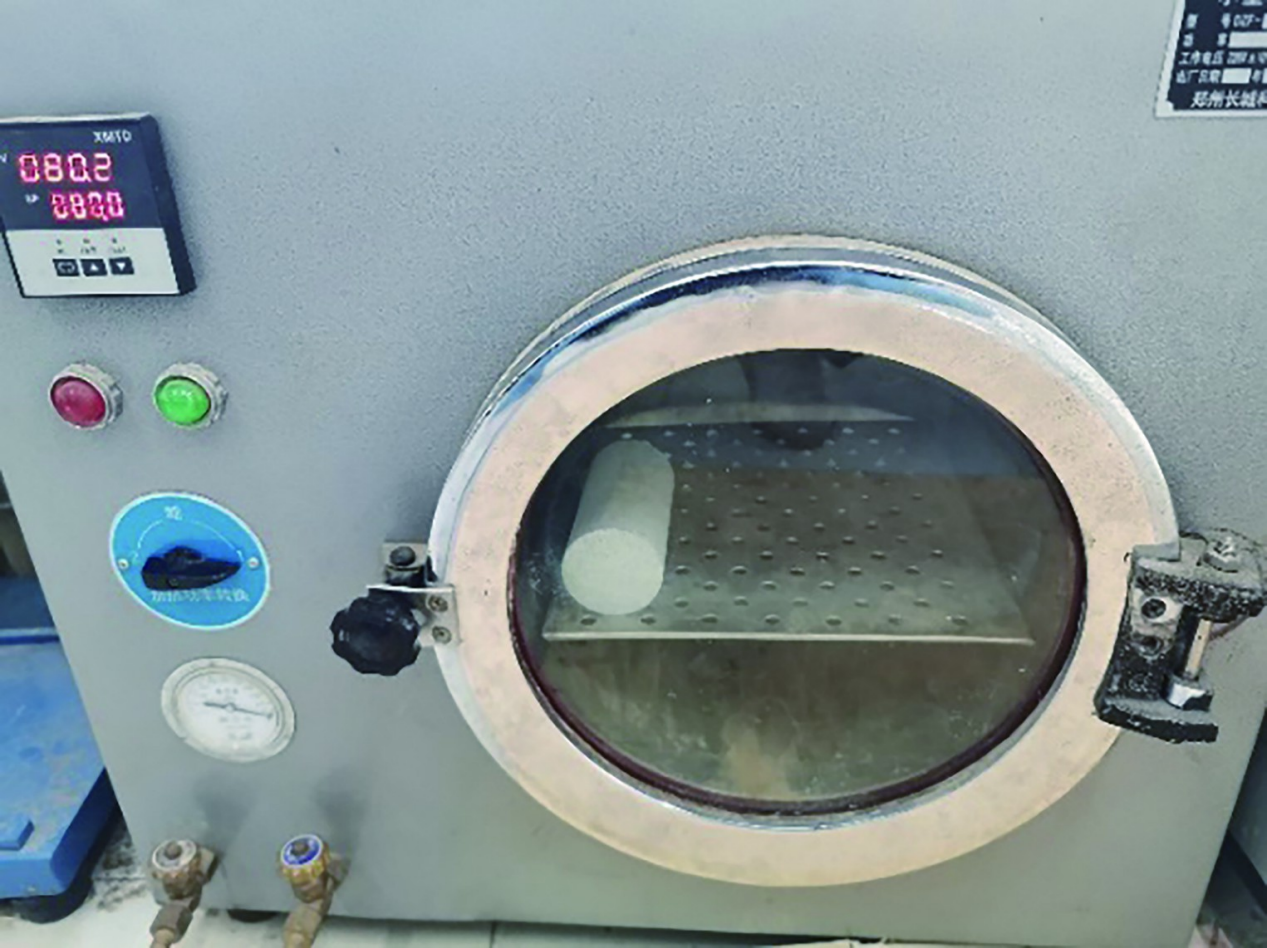
Specimen heating box.

**Fig 13:**
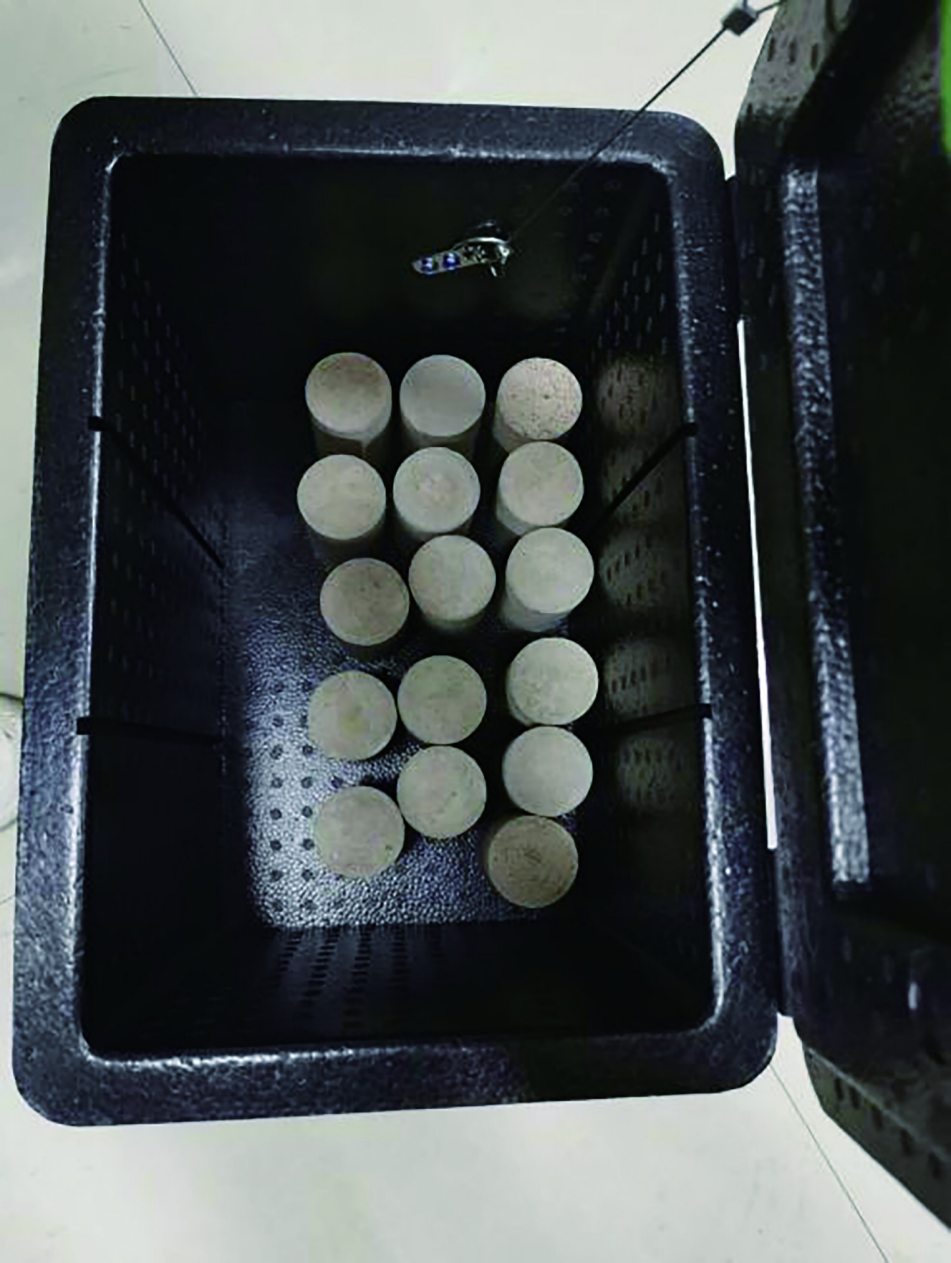
Placement arrangement of specimens in an incubator.

**Fig 14:**
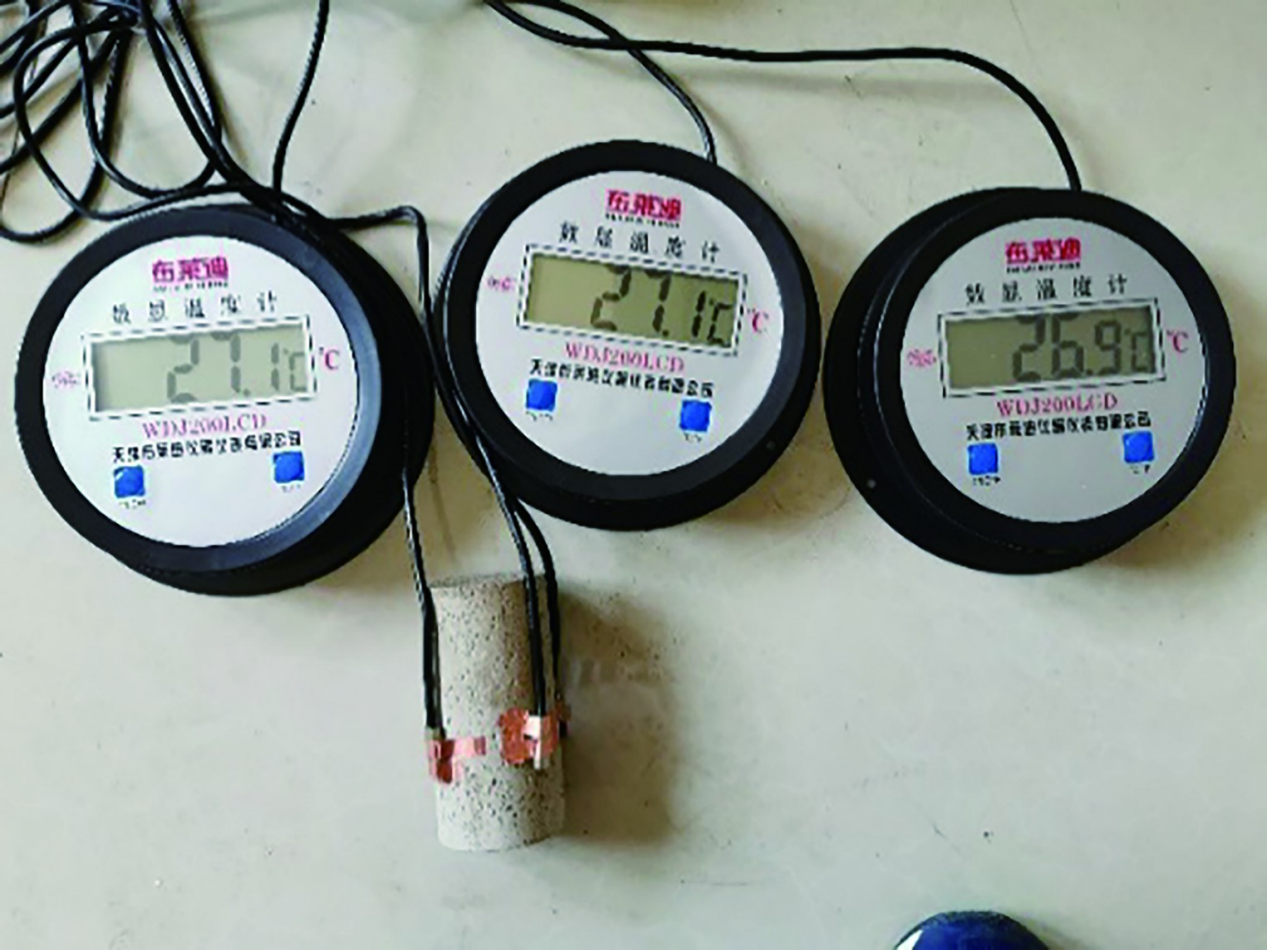
Specimen temperature measuring devices.

**Fig 15:**
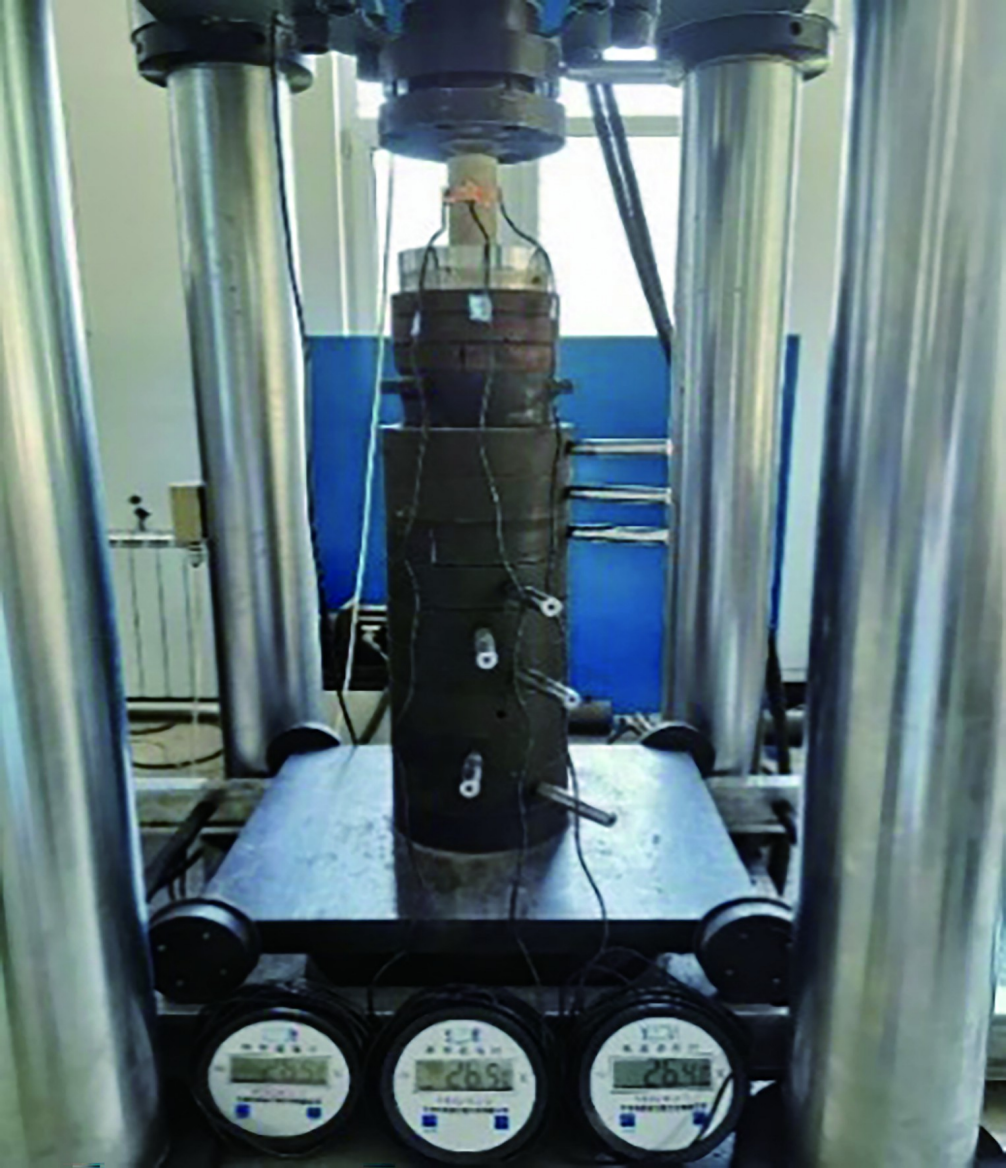
Test installation.

**Fig 16:**
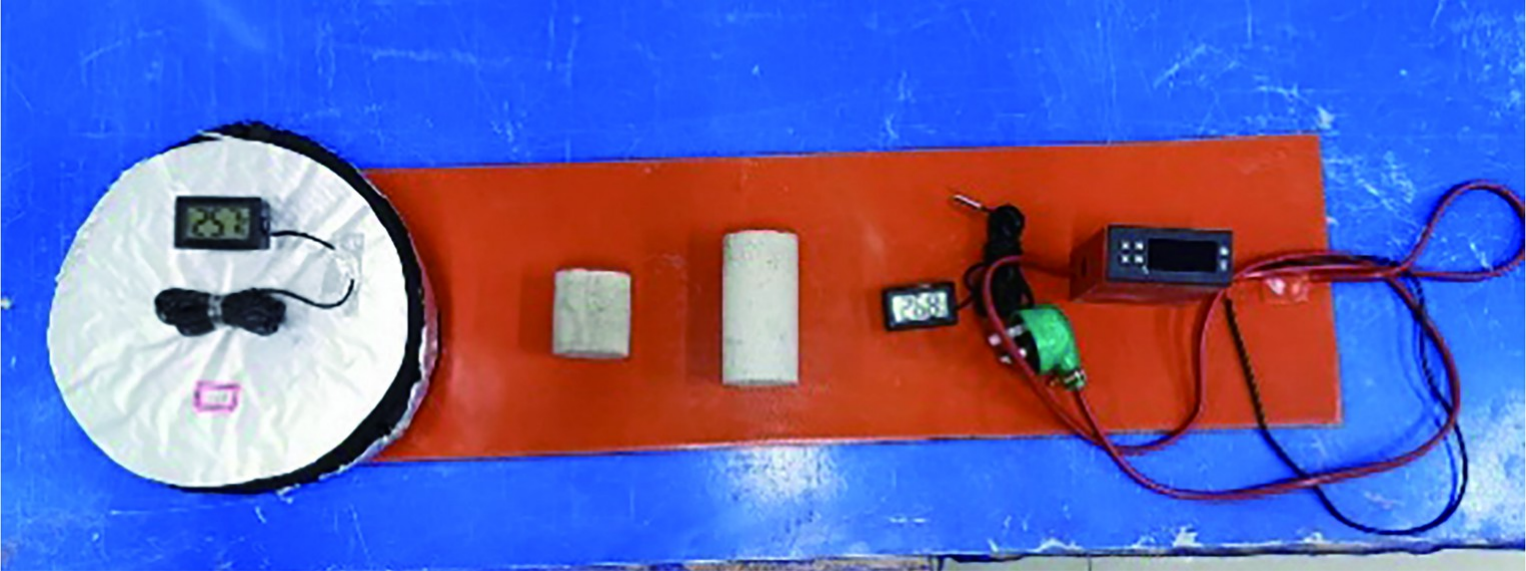
Heating plate used during the experiment.

**Fig 17:**
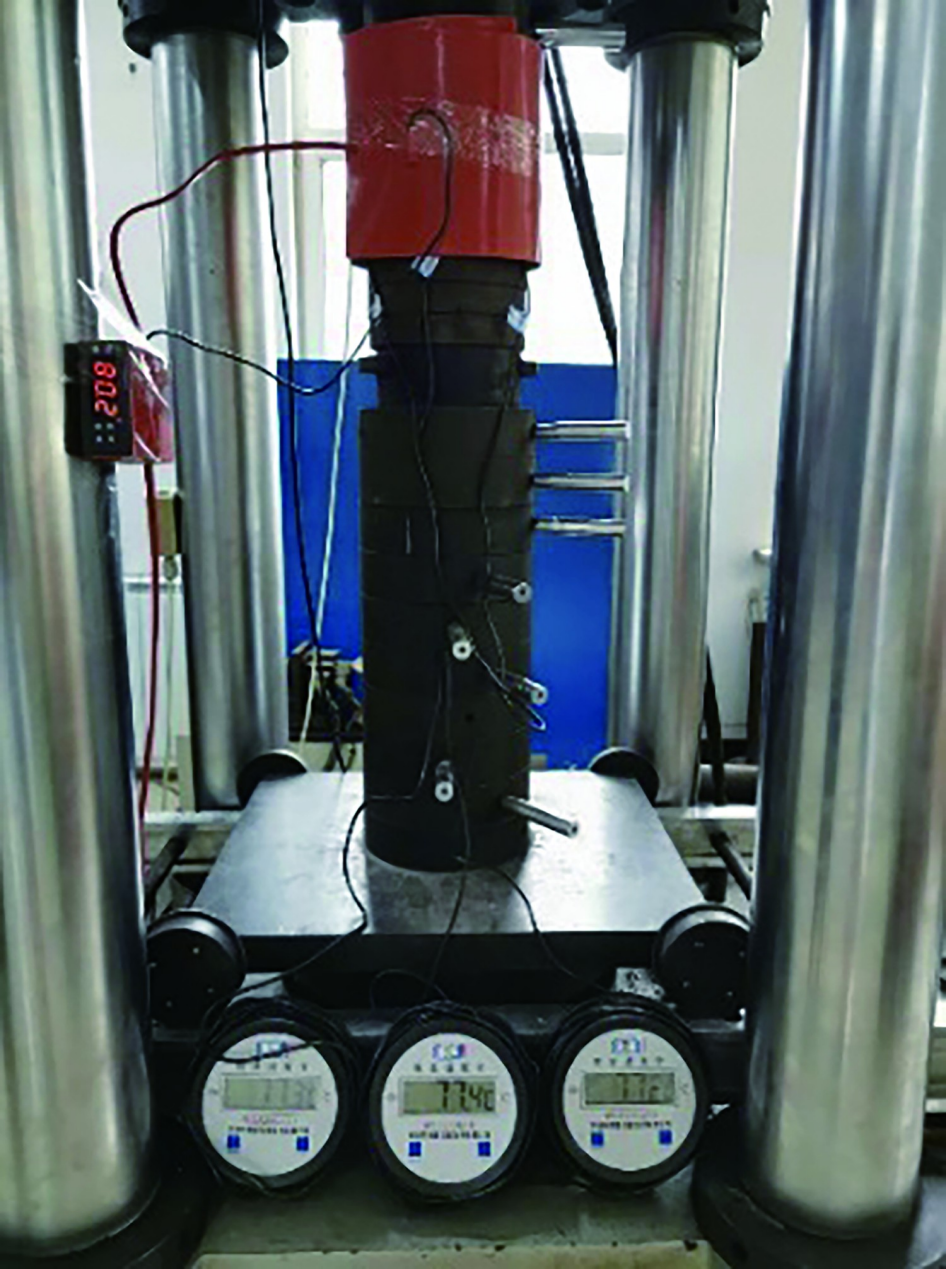
Photograph showing the experimental procedure setup.

1. The rockburst-like material test pieces were divided into six groups, placed in the heating box in six batches, heated to a predetermined temperature, and allowed to stand for 2 h, as shown in Fig 12.
2. A plastic incubator was prepared in advance to avoid temperature changes of similar material models, as shown in Fig 13.
3. By wearing heat-resistant gloves and using high-temperature-resistant tape, the thermometer’s temperature-sensing patch was quickly attached to the test piece. The sensor line extended outside the uniaxial compression testing machine and the thermometer was placed on the outside to prevent damage to the digital display thermometer and sensing line when the specimen was compressed. The thermometer was placed on the test machine and the three thermometers were marked as 1, 2, and 3, respectively, as shown in Fig 14. The test piece was placed on the testing machine, as shown in Fig 15; the temperatures displayed by the three thermometers of the test piece were noted and their average value was taken.
4. The structure of the constant temperature heating plate is shown in Fig 16. The heating plate was placed on the testing machine and bent with the sticky side facing inward. The upper part of the heating plate and the upper part of the testing machine were pasted; the light-emitting diode (LED) screen of the heating plate displayed its own temperature. Some space was left between the lower part of the heating plate and the steel block to ensure that the testing machine was not disturbed when it rose. The heating plate comes with a temperature probe. This probe was placed into the sleeve outside the heating plate to detect the temperature of the heating plate. We waited for the temperature to rise to a stable state, and subsequently, for an additional 10–20 min to allow the formation of a constant temperature field between the heating plate and the specimen. At this point, we paid close attention to the temperature displayed on the thermometer and the on-screen temperature displayed on the heating plate.
5. According to the original temperature of similar material specimens, we set the temperature of the heating plate to be slightly higher than that of the model. After the temperature in the heating plate stabilized, the temperature was maintained for a period of time to ensure that the temperature of the specimen and the heating plate was stable at the expected temperature. Subsequently, the uniaxial compression test was initiated. Loading was conducted at a speed of 0.1 MPa/s until the sample failed, as shown in Fig 17.
6. After the test, the power of the thermostat was turned off, the heating plate was removed and cooled to 25°C, and subsequently set it in place. Next, the test piece was removed, the high-temperature resistant tape was teared off, and the heating plate was removed.

#### Variation of brittleness and energy of similar materials at different temperatures

According to the energy theory, the occurrence of rockbursts can also be predicted and evaluated. The maximum elastic strain energy of the rock mass is defined by the following formula:

$$E_{s}=\sigma_{c}^{2}/(2E),$$ (8)

where E is the elastic modulus of the rock (GPa), σ_c_ is the uniaxial compressive strength of the rock (MPa), and E_s_ is the maximum stored strain energy of the specimen (MJ/m^3^).

**Fig 18:**
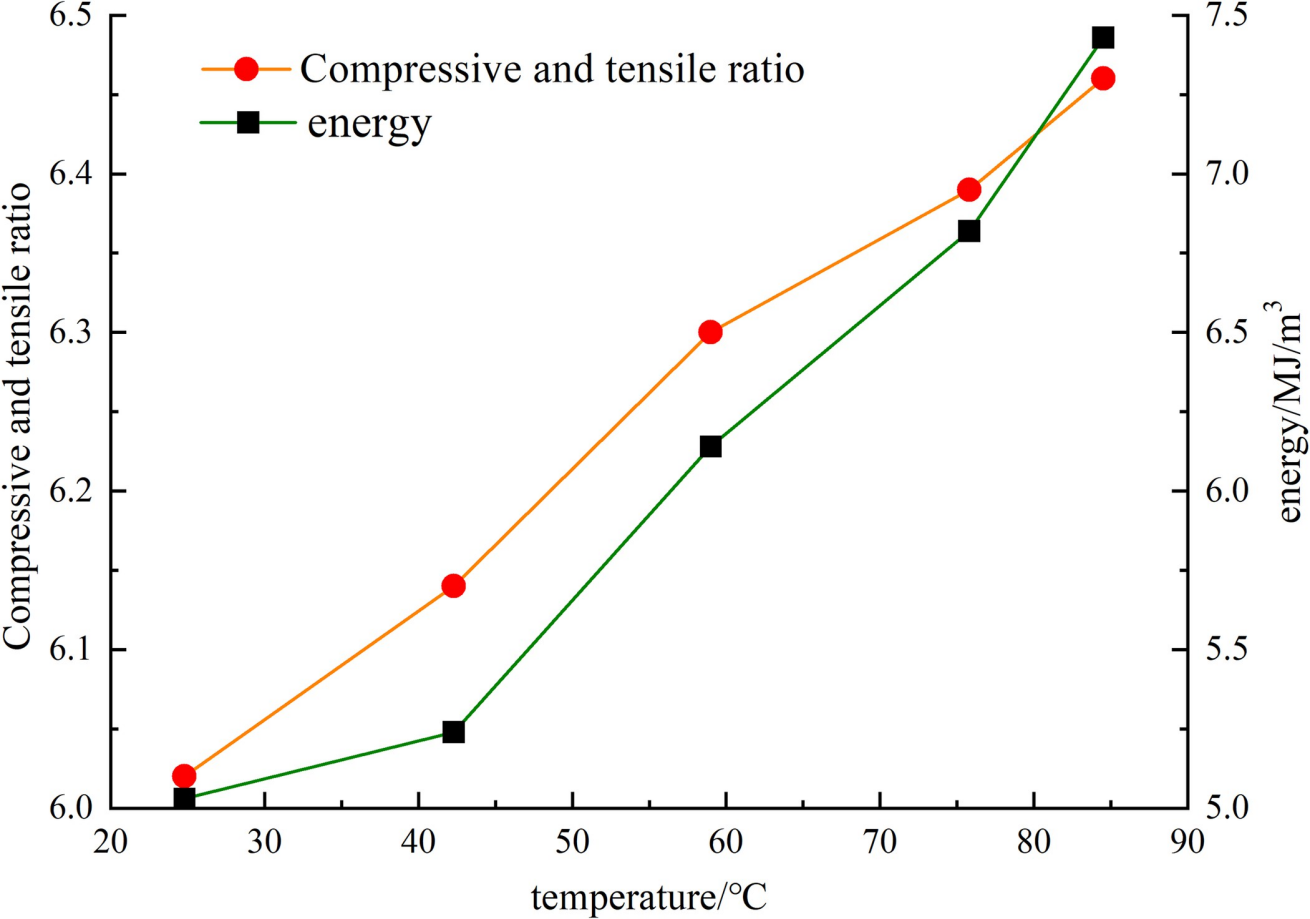
Variation of brittleness and energy of similar materials at different temperatures covered by the study.

The brittleness and energy changes in rockburst similar materials at different temperatures were calculated, and the results are shown in Fig 18.

From the experimental data for brittleness and energy of the model in Fig 18, it can be seen that as the temperature increases, the brittleness of the similar materials gradually increases, and the maximum elastic strain energy of the specimen increases accordingly. From the above calculation and analysis, it can be observed that when the rockburst similar material initially fails, the small cracks inside the specimen continue to extend with the application of pressure, and the excess energy is transformed into kinetic energy. Under the action of temperature, the distance between the crystals inside the rock mass increases, and the internal voids of the rock mass increase owing to dislocation slippage. Under the coupling effect of temperature and stress, the damage and destruction of the internal basic structure of the test piece are exacerbated. According to the rockburst observation data, it can be concluded that the process of rockburst essentially involves accumulation of energy in the early stage and its release under different conditions in the later stage. The process can be divided into the three stages of energy accumulation, microcrack formation and propagation, and crack penetration and bursting.

### Comparative analysis of temperature effects between white sandstone and similar materials

**Fig 19:**
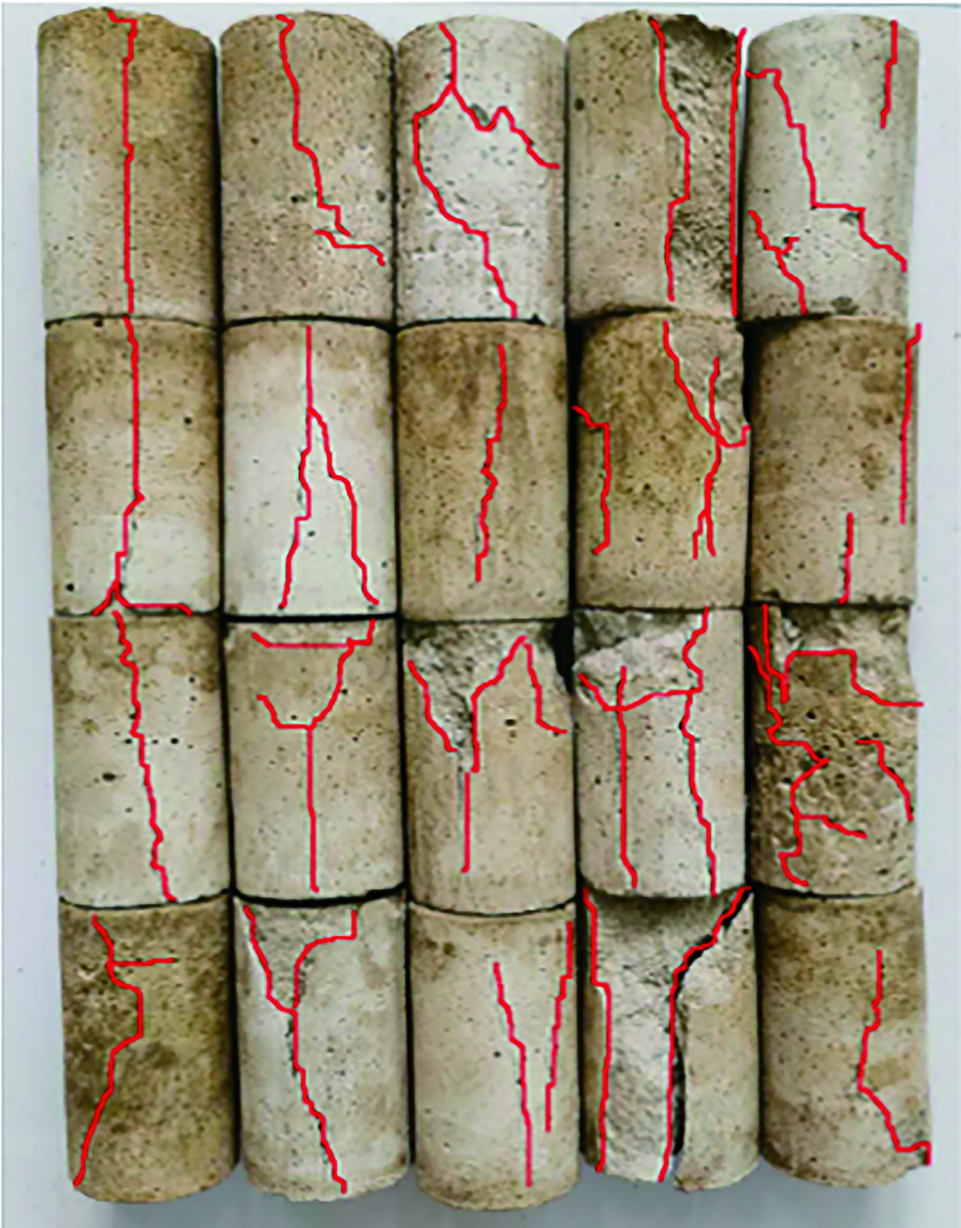
Photograph showing similar material failure patterns.

**Fig 20:**
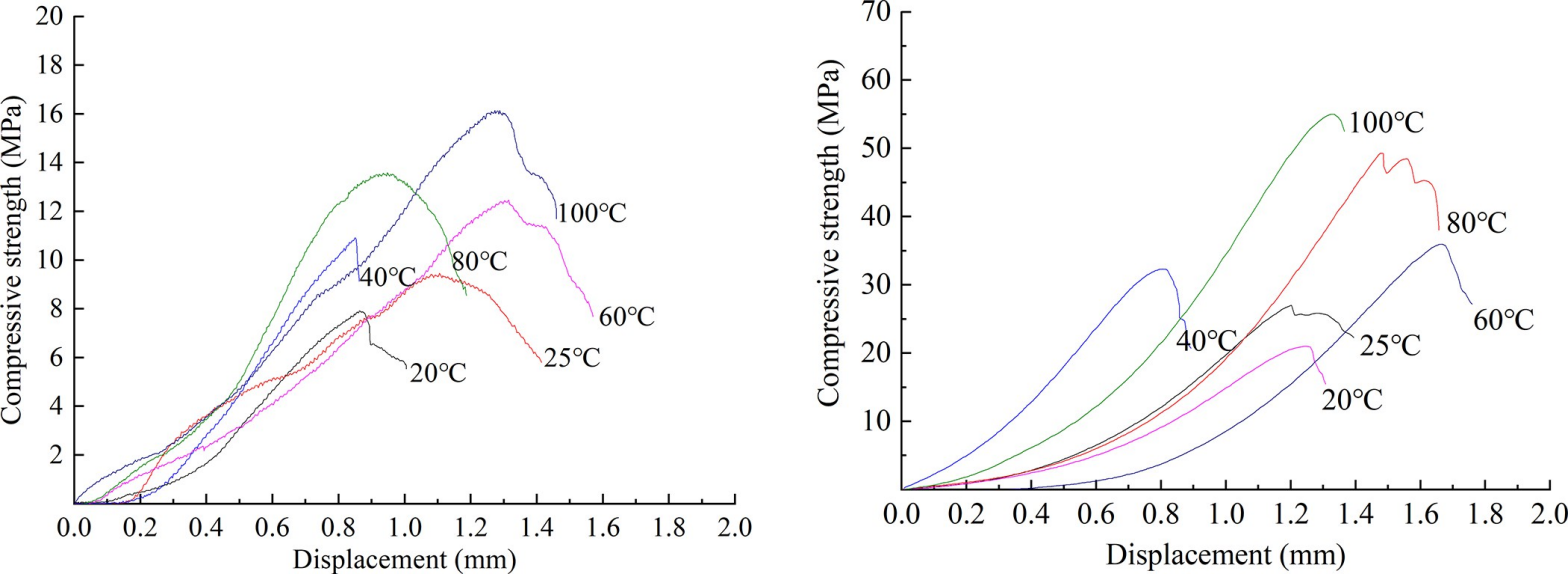
Stress–displacement curves of typical specimens at different temperatures. (a) Compressive strength responses of similar materials. (b) Raw rock.

The uniaxial compression test results of similar materials are shown in Fig 19. By analyzing the test data of the uniaxial compressive test of similar materials, the stress–displacement curves of the six groups of typical similar materials subject to the temperature effects are shown in Fig 20(A).

The uniaxial compressive strength test of the original white sandstone rock was conducted by the same test procedure as that described in Section 3.2. The stress–displacement curves of the six groups of original white sandstone rock subject to the condition of the temperature effect are shown in Fig 20(B).

The stress–displacement curves of rockburst-like materials and original rock at different temperatures can be divided into the following four stages. First stage: the initial compaction stage. Herein, due to the action of external force, the existing microcracks in the rock body were compressed. Second stage: the elastic deformation stage. Herein, the rock was deformed subject to the action of external force; the deformation could be recovered when the external force was removed. As the displacement of the rock particles breaks the original internal stability and equilibrium, the rock particles gain some of their energy. Within a very short time, this potential energy occurs by eliminating external forces, so that the displacement of the particle returns to its original place, a phenomenon known as elastic deformation recovery. The third stage: the plastic deformation stage. Herein, the curve exhibited upward concave characteristics and the slope of the curve gradually became smaller. The slopes of the upward concave curves of rockburst-like materials and white sandstone at different temperature conditions were different; the slope of the curve became zero when the stress peak was reached. Owing to the brittleness of rockburst-like materials and white sandstone, the plastic deformation of the specimen was extremely small. When plastic deformation occurred, the particles inside the rock slipped and deformed. In addition, a pressure-solution recrystallization of the rock subject to the action of strong stress was a mechanism for the plastic deformation of the rock. Fourth stage: the destruction stage. Herein, the curve dropped rapidly and the slope of the curve assumed a negative value. When the external force applied to the rock exceeded the strength limit that the rock can bear, the internal structure of the rock was damaged; this resulted in a fractured surface.

**Fig 21:**
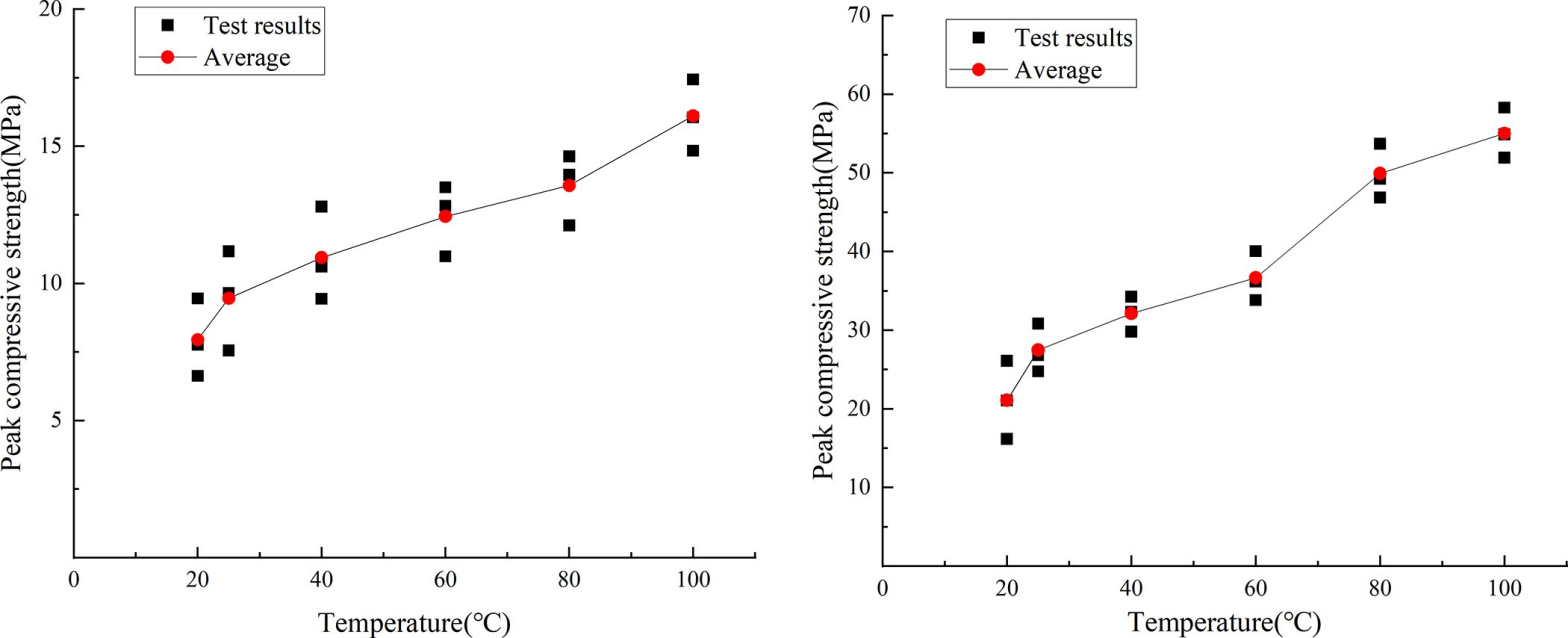
Curve of peak compressive strength at different temperatures. (a) Compressive strength responses of similar materials. (b) Raw rock.

Fig 21(A) shows the curve of the peak uniaxial compressive strength of the similar material model of white sandstone at different temperature conditions as a function of temperature. The peak uniaxial compressive strengths of similar materials increased as a function of temperature. The strength was 19.47, 15.42, 14.09, 8.98, and 18.76% higher at 25, 40, 60, 80, and 100°C than that at 20, 25, 40, 60, and 80°C, respectively. These changes are attributed to the internal porosity of rock, which has a great influence on the uniaxial compressive strength. With the increase in temperature, the cohesiveness of the cementing material in the rock changed, the pore structure in the rock tended to be stable, and the influence of porosity on similar material models gradually increased.

Fig 21(B) shows the changed curve of the peak uniaxial compressive strength of white sandstone original rock with temperature at different temperature conditions. The peak uniaxial compressive strength of the original white sandstone rock increased as a function of temperature. The strength was 33.00, 14.81, 12.90, 45.71, and 6.27% higher at 25, 40, 60, 80, and 100°C than that at 20, 25, 40, 60, and 80°C, respectively. The reason behind these trends is that owing to thermal expansion, the cohesion between the particles in the white sandstone is weakened and the moisture in the particles is reduced. This results in an increase in the brittleness of the rock and an increase in the compressive strength.

By comparing the peak compressive strength curves of rockburst-like materials and white sandstone at different temperatures, it can be observed that the variation trend of the peak stress curves of rockburst-like materials and raw white sandstone rock is highly consistent during the process of uniaxial compression; both curves exhibited increasing trends. Furthermore, both of them are attributed to the thermal expansion effect of the rock, which became gradually obvious as the temperature increased. Additionally, the peak stress gradually increased.

**Fig 22:**
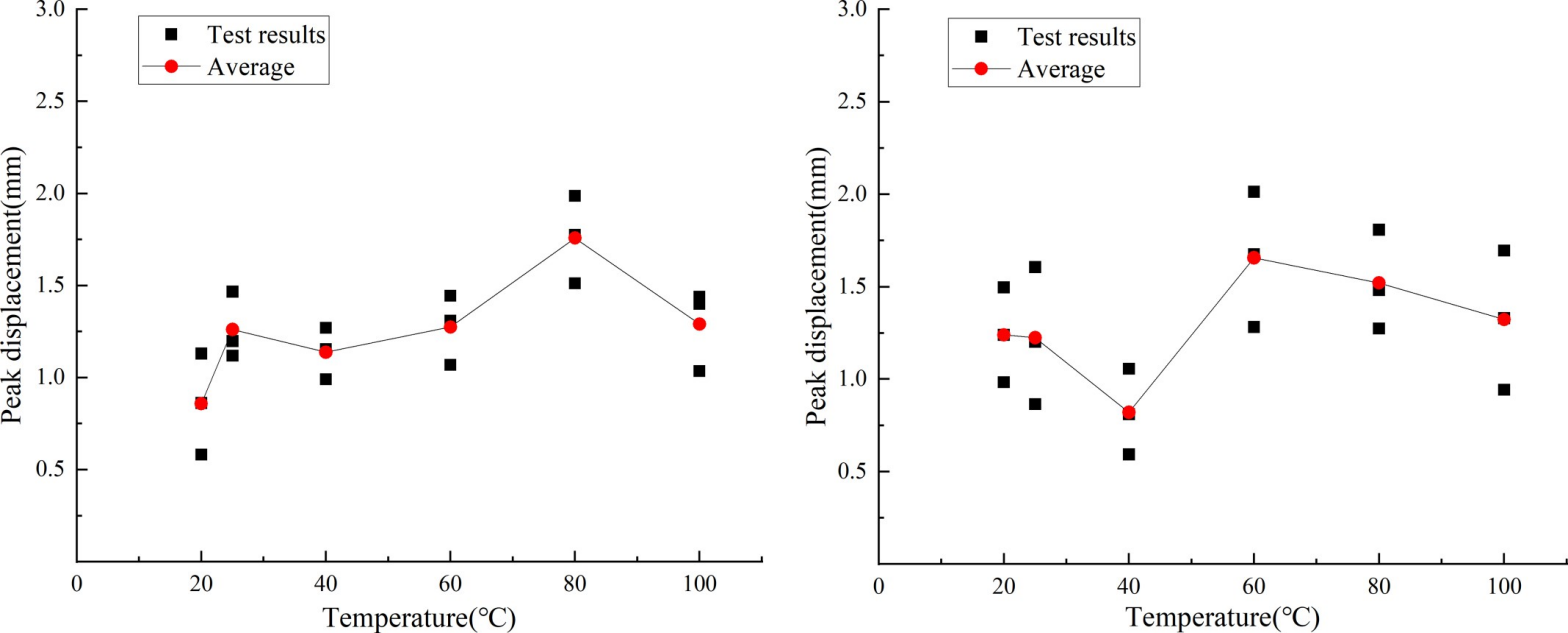
Peak displacement curve at different temperatures. (a) Compressive strength responses of rockburst-like materials. (b) Raw rock.

Fig 22(A) shows the vertical displacement curve of the similar material model of white sandstone when it reaches the peak compressive strength at different temperature conditions. At 25°C, it decreased by 9.15%; at 60 and 40°C, it increased by 15.1 and 33.46% compared with 40 and 60°C, respectively; and at 100°C it decreased by 26.63% compared with 80°C. From the above-mentioned data, it can be observed that the vertical displacement of similar material models of white sandstone exhibit no particular trends as a function of temperature. The vertical displacements of similar material models reach their maxima when the temperature is 80°C, and the vertical displacement of similar material models is minimized when the temperature is 20°C. Because of the thermal expansion and cold contraction effect, the higher the temperature, the higher the pressure in the rock pores. The compaction between the various crystals is gradual, which makes the rock mass exhibit obvious brittle characteristics.

Fig 22(B) shows the vertical displacement curve of the original white sandstone when the compressive strength reaches the peak value at different temperature conditions. It can be observed from the fig that at 25°C, the vertical displacement is 0.40% lower than that at 20°C, and at 40°C, it is lower than that at 25°C. The strength decreased by 35.74%, at 60°C, increased by 95.12% compared with that at 40°C. At 80 and 100°C it decreased by 5.00 and 14.47% compared with that at 60 and 80°C, respectively. When the temperature was 60°C, the vertical displacement value of the white sandstone original rock was the largest, and when the temperature was 40°C, the vertical displacement value was the smallest. This is because the original rock itself had a certain hardness and brittleness. Therefore, when the temperature was in the range of 40–60°C, the vertical displacement of white sandstone exhibited a sharp increasing trend. Above 60°C for rocks, the hardness and brittleness of rocks gradually transitioned to ductility. This led to a downward trend in the vertical displacement.

By comparing the variation curves of peak displacement of rockburst similar material and raw rock at different temperatures, it can be observed that in the process of uniaxial compression, the variation trends of peak displacement curves of rockburst similar material and raw rock are different, but both of them exhibit an increasing followed by a decreasing trend. This difference is attributed primarily to the difference of cementation between grains, porosity between grains, hard brittleness, and peak stress between the two, thus resulting in different vertical displacement changes of the two when the temperature changes.

**Fig 23:**
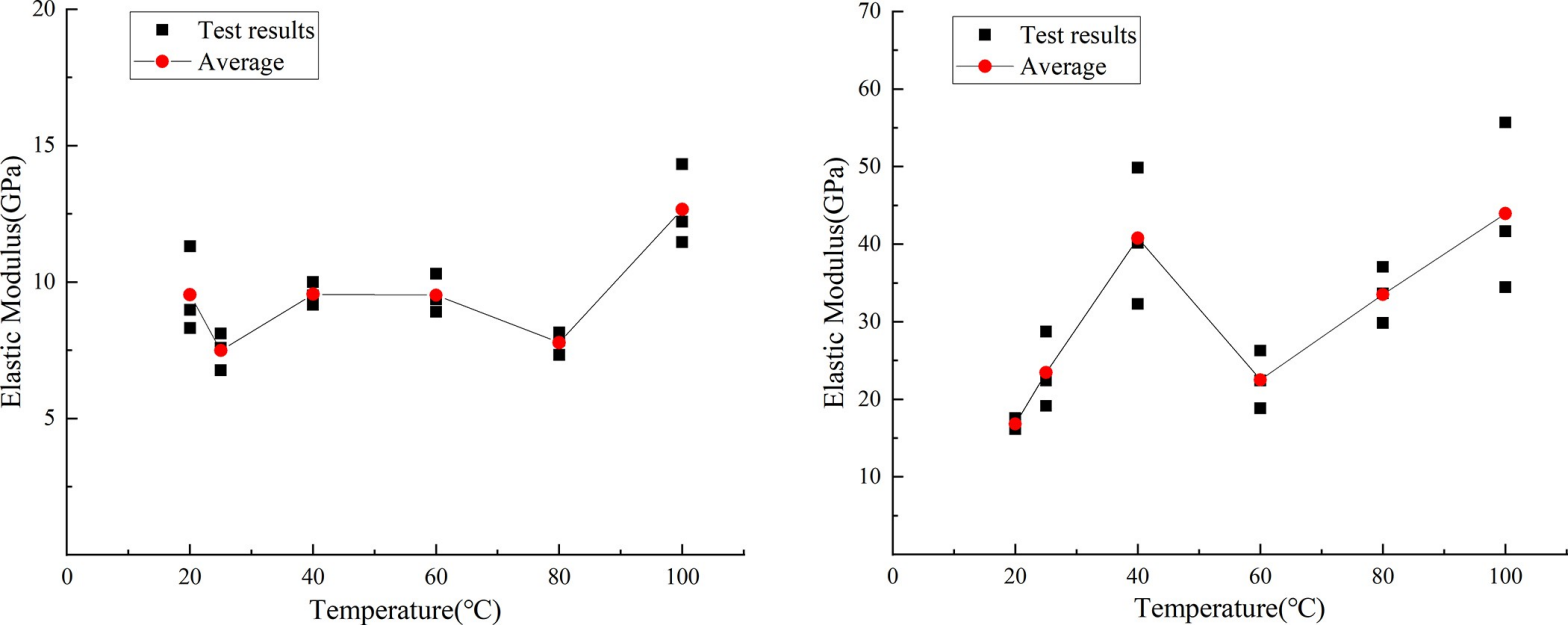
Elastic modulus curve at different temperatures. (a) Elastic moduli responses of rockburst-like materials. (b) Raw rock.

Fig 23(A) shows the change of elastic modulus of similar white sandstone materials at different temperature conditions. It can be observed from the figure that the modulus at 25°C was 18.05% lower than that at 20°C; at 40°C, it was 27.03% higher than that at 25°C; at 60 and 80°C, it was 0.08 and 18.34% lower than that at 40 and 60°C, respectively; and at 100°C it was 61.89% higher than 80°C. The elastic modulus increases as the temperature increases. The minimum (7.53 GPa) and maximum (12.54 GPa) value of the elastic modulus appeared at 25 and 100°C, respectively. At 25°C, the crystal structure yielded only small changes and the molecules in the rock did not move considerably. The model exhibited low strength and high-brittleness characteristics. At 100°C, owing to the excessively high temperature, the plasticity of the rock increased and the cohesion became larger.

Fig 23(B) shows the change of elastic modulus of white sandstone at different temperature conditions. At 25 and 40°C, the modulus increased by 43.75 and 69.57% compared with that at 20 and 25°C, respectively. At 60°C, it increased by 69.57%, and it was 41.03% lower than that at 40°C. At 80 and 100°C, it was 39.13 and 25.00% higher than that at 60 and 80°C, respectively. In the range of 20–100°C, the maximum and minimum value of the elastic modulus appears at 40 and 20°C, respectively. When the temperature is in the ranges of 20–40°C and 60–100°C, the white sandstone particles have strong bonds and high-elastic moduli. However, at 40–60°C, the elastic modulus shows a significant decline owing to the fact that the cement between particles is destroyed at high temperatures. Once the external force is eliminated, the rock is difficult to recover to the previous state, and the elastic modulus value decreases.

By comparing the variation curves of elastic modulus of rockburst-like material and white sandstone at different temperatures, it can be observed that the variation trends of elastic modulus curves of rockburst-like material and white sandstone are different during the process of uniaxial compression. The main reason for this is that rockburst-like materials and original white sandstone rock have different cementation, porosity, hardness and brittleness, peak stress, and elastic modulus characteristics. However, they exhibit the same stages because they both have a tendency to transition from brittleness to ductility as the temperature changes.

### Analysis of rock damage characteristics at different temperatures

**Fig 24:**
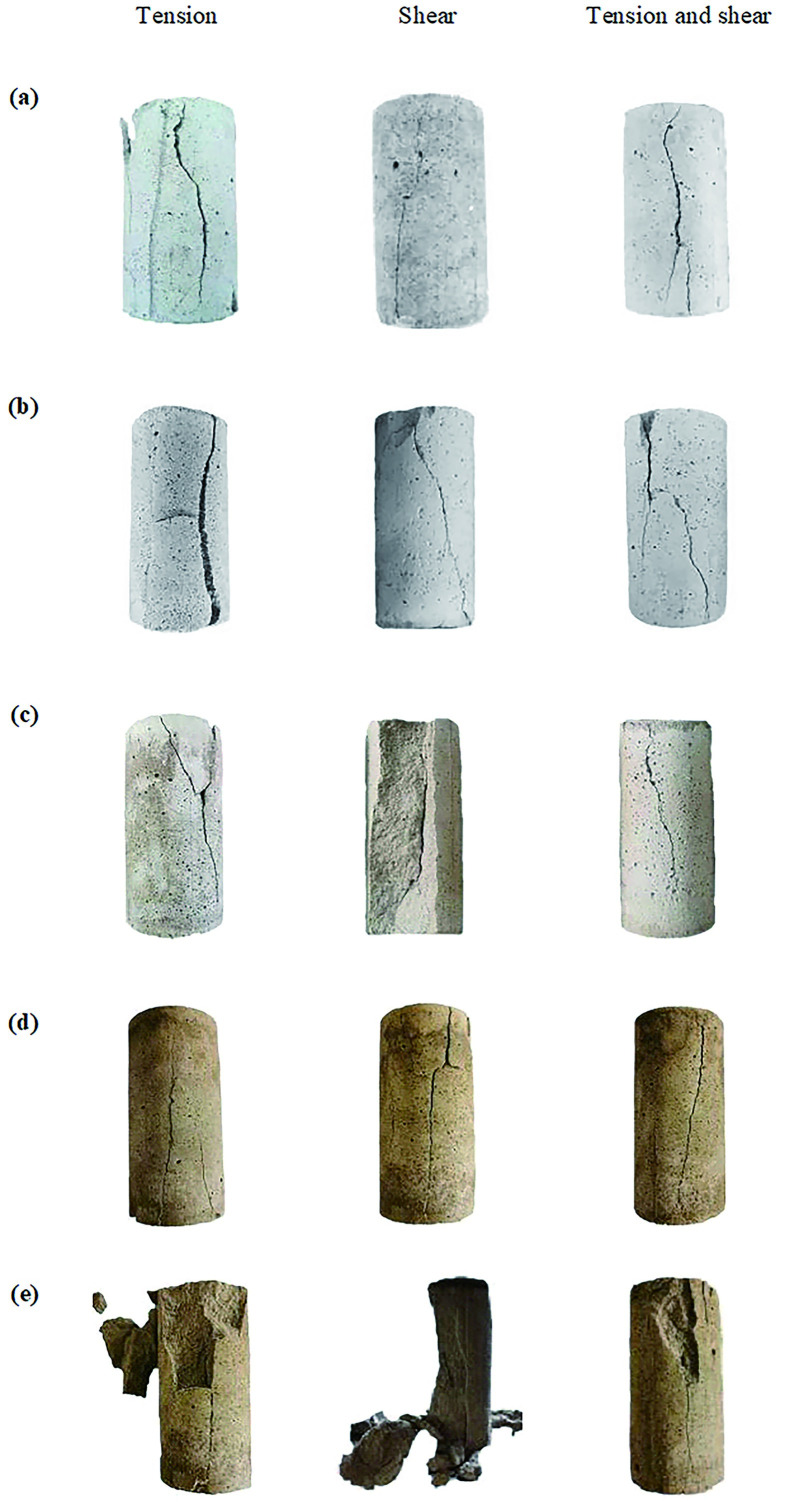
Failure mode of the test piece for rockburst-like material. (a) 20°C. (b) 40°C. (c) 60°C. (d) 80°C. (e) 100°C.

**Fig 25:**
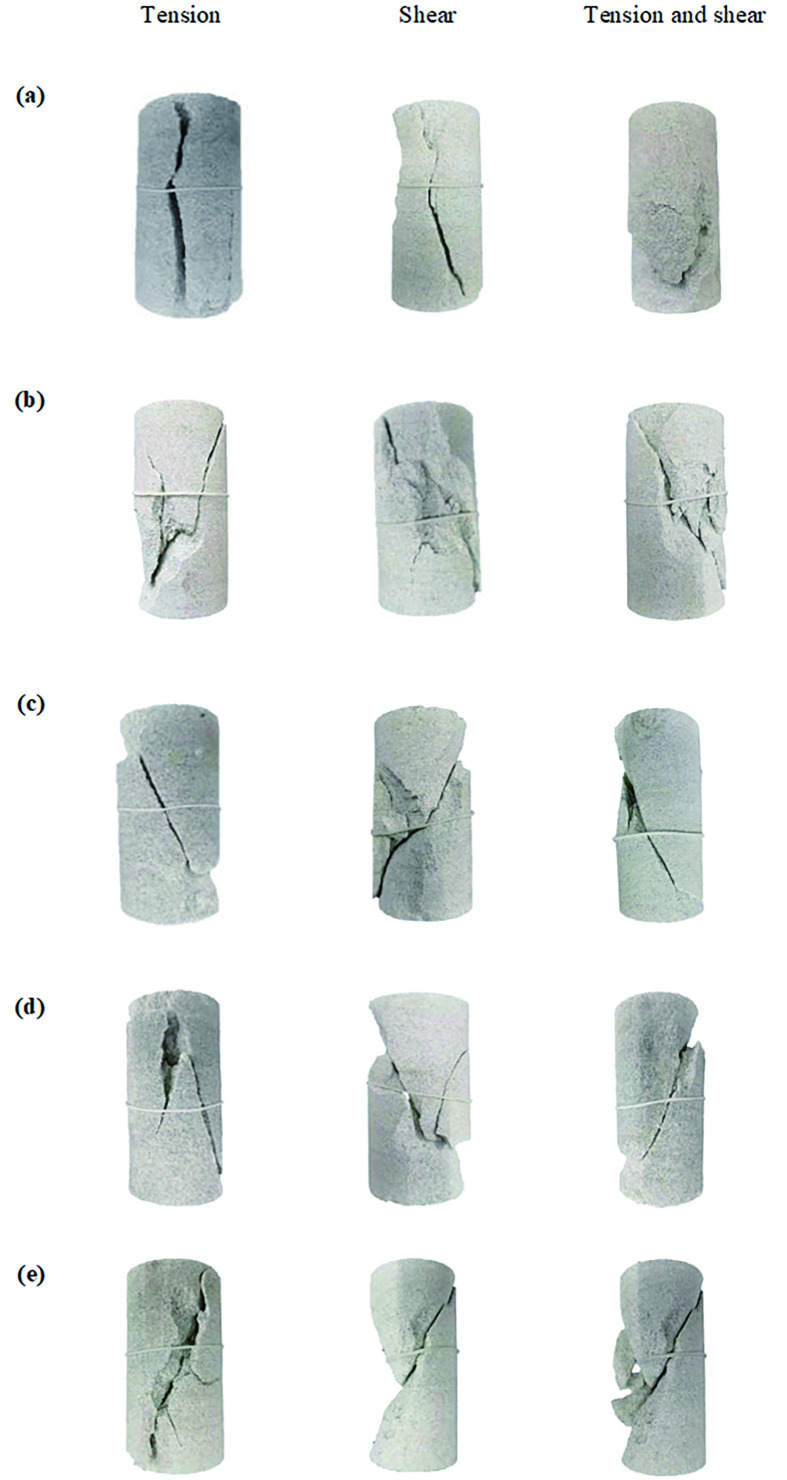
Failure mode of the test piece for raw rock. (a) 20°C. (b) 40°C. (c) 60°C. (d) 80°C. (e) 100°C.

Comparing and analyzing the failure modes of original rock specimens and similar rockburst materials, it is inferred that few specimens exhibit tension and shear failure, and most specimens exhibit tension and shear-composite failures. The failure modes of the specimens are shown in Figs 24 and 25.

It can be observed from Fig 24 that at 20°C, the failure mode of the similar material model of white sandstone is an axial vertical splitting failure with cracks running from top to bottom, and derivative small cracks rarely appear around the cracks. When the temperature rises to 40°C, the failure mode of similar materials is primarily tensile failure, which is generally accompanied by the appearance of shear cracks. When the temperature rises to 60°C, white-sandstone-like materials are primarily in the form of tension-shear composite failure. In the form of composite failure, tensile and shear cracks usually appear simultaneously. In general, the tensile cracks appear around the main shear crack. When the temperature rises to 80°C, the failure mode of similar materials is primarily a composite failure mode, multi-angle tensile failure cracks appear around the main shear crack, and some areas of the model will be broken and dropped. When the temperature rises to 100°C, all white sandstone-like material models are in the form of tension–shear composite failure. After the failure of the model, a discontinuous main shear crack appears through the model and a relatively loose fracture zone is formed around the main shear crack, thus forming an obvious shear zone.

From the comparison between the original white sandstone and similar materials in Figs 24 and 25, it can be observed that the failure mode of the similar material is the same as that of the original rock, and a similar material can be a good substitute for the original white sandstone for further experimental research.

Based on the comparative analysis of the failure forms of rockburst-like materials and original rock, it can be observed that during the uniaxial compression test, there are three changes in the particles in the rock body, namely, elastic deformation, fissure slip, and pore closure. The frictional force inside the rock mass has different effects owing to changes in temperature and stress, thus resulting in different rock failure characteristics. The existence of stress increases both the cracks in the rock and the axial deformation of the rock specimen such that the rock has a smaller elastic modulus. With increase in temperature, the color of the rockburst-like materials gradually deepened, but the color of the original rock did not change considerably. This is attributed to the difference in the crystal structure and density of the rock, thus resulting in the difference in the physical properties of the two.

Subject to the action of stress, the external energy acting on the specimen was relatively small and the internal, random, disordered microcracks developed in an orderly direction. The crack development was not sufficient; additionally, only a small number of cracks were connected, the number of broken pieces was small, and sometimes there was no macroscopic failure phenomenon. As the stress increased, the energy which acted on the specimen increased and the cracks in the specimen fully developed, continuously grew, and became interconnected to form macrocracks. The number of new cracks was high, number of fragments was increased, size of fragments was reduced, and the degree of fragmentation was intensified.

## Conclusions

1. Based on the white sandstone, the ratio of similar materials was optimized. The results showed that the optimal ratios of white-sandstone-like materials were: quartz sand content of 36%, iron powder content of 1.9%, gypsum–cement ratio of 1.8:1, and sand particle size in the range of 2–4 mm. The evaluations of the rockburst tendency revealed that similar materials had strong rockburst tendency, low strength, and high-brittleness characteristics
2. Based on the evaluations of stress, displacement, and elastic modulus, the major findings were as follows. In the temperature range of 20–100°C, the compressive strength of rockburst-like materials increased gradually as a function of temperature. Whereas, the vertical displacement first increased and then decreased as a function of temperature. The minimum and maximum value of elastic modulus appeared at 20 and 100°C, respectively
3. The stress–strain curves of similar rockburst materials and original rock at different temperatures were divided into four stages: initial compaction, elastic deformation, plastic deformation, and failure
4. Based on the comparative analysis of the failure forms of rockburst-like materials and original rock, it was observed that during the uniaxial compression test, there were three changes in the particles in the rock body, namely: elastic deformation, fissure slip, and pore closure
5. Comparing the failure modes of original rock specimens and rockburst-like materials, it was shown that a few specimens exhibited tensile and shear failure, and most specimens exhibited a combination of tension and shear failure

## Supporting information

S1 Fig Influences of various factors on compressive strengths of rockburst-like materials. (OPJU)

S2 Fig Influences of various factors on tensile strengths of rockburst-like materials. (OPJU)

S3 Fig Influences of various factors on the elastic moduli of rockburst-like materials. (OPJU)

S4 Fig Influences of various factors on the compressive tensile ratios of rockburst-like materials. (OPJU)

S5 Fig Evaluations of rockburst tendencies based on compression and tensile ratio. (OPJU)

S6 Fig Determination of rockburst tendencies based on the area ratio before and after peak. (OPJU)

S7 Fig Variation of brittleness and energy of similar materials at different temperatures covered by the study. (OPJU)

S8 Fig Stress–displacement curves of compressive strength responses of similar materials specimens at different temperatures. (OPJU)

S9 Fig Stress–displacement curves of raw rock at different temperatures. (OPJU)

S10 Fig Curve of peak compressive strength of compressive strength responses of similar materials at different temperatures. (OPJU)

S11 Fig Curve of peak compressive strength of raw rock at different temperatures. (OPJU)

S12 Fig Peak displacement curve of compressive strength responses of rockburst-like materials at different temperatures. (OPJU)

S13 Fig Peak displacement curve of raw rock at different temperatures. (OPJU)

S14 Fig Elastic modulus curve of elastic moduli responses of rockburst-like materials at different temperatures. (OPJU)

S15 Fig Elastic modulus curve of raw rock at different temperatures. (OPJU)
